# Robust Amorphous MOF‐Based Aerogels Digital Biosensor for Sensitive Detection of Organophosphate Pesticides

**DOI:** 10.1002/advs.75997

**Published:** 2026-06-09

**Authors:** Changshun Su, Xiangyu Zhai, Houru Li, Yijie Wang, Hongxia Li, Yueyao Jiang, Geyu Lu, Xu Yan

**Affiliations:** ^1^ Department of Food Quality and Safety College of Food Science and Engineering Jilin University Changchun P. R. China; ^2^ State Key Laboratory on Integrated Optoelectronics Key Laboratory of Advanced Gas Sensors of Jilin Province College of Electronic Science & Engineering Jilin University Changchun P. R. China; ^3^ Department of Pharmacy China–Japan Union Hospital of Jilin University Changchun P. R. China

**Keywords:** aerogel digital biosensor, amorphous MOF, enzyme, POC test

## Abstract

Enzyme‐based point‐of‐care (POC) biosensors offer rapid on‐site detection capabilities; however, their practical deployment is fundamentally constrained by limited sensing stability. Enzyme immobilization strategies enhance sensing stability but often compromise the catalytic activity of enzyme, thereby constraining the POC biosensor performance. In this study, a robust aerogel digital biosensor (ADB) was constructed by embedding enzyme‐mediated amorphous MOFs (AChE‐AMOF) into a calcium (II)/alginate aerogel system for stable and precise on‐site detection of organophosphate pesticides. The enzyme‐mediated process perturbs the Zn^2+^ coordination environment and disturbs the orderly MOF‐74 framework, producing a porous AChE‐AMOF composite that facilitates substrate accessibility and mitigates conformational changes of the immobilized enzyme. Impressively, the amorphous AChE‐AMOF exhibits a 2.2‐fold higher catalytic activity than AChE loaded in the crystalline MOF structure. The combination of the AMOF architecture and the aerogel's porous network confers high stability on the ADB, enabling it to retain over 98% of its enzymatic activity for 50 days. Moreover, the output signal of ADB can be converted into digital information by an image algorithm, enabling precise quantification of paraoxon at the nanogram per millilitre level. This work provides a robust tool for accurate on‐site pesticide detection and offers meaningful insights into the design of high‐performance enzyme‐based POC biosensors.

## Graphical abstract

We report a robust aerogel digital biosensor by integrating an enzyme‐mediated amorphous MOF within an aerogel matrix for on‐site pesticide detection. The enzyme‐mediated assembly strategy enables in situ tailoring of MOF into a porous amorphous structure, achieving both high stability and activity. The aerogel matrix further enhances enzyme stability and practical performance, enabling detection of pesticides at the nanogram‐per‐milliliter level.

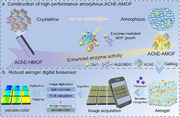

## 1 Introduction

Point‐of‐care (POC) detection of pesticides is crucial to safeguard human health and food safety by enabling rapid on‐site screening and early identification of hazardous residues [1, 2, 3]. Conventional POC platforms, including colorimetric assays, lateral flow immunoassays, and portable electrochemical sensors, offer simplicity and rapid signal readout [2, 4, 5]. However, their robustness is limited by the susceptibility of biological recognition elements (e.g., enzymes) to denaturation under external stimuli, leading to signal drift and reduced detection reliability [6, 7]. Enzyme immobilization within biocompatible carriers provides an effective strategy to enhance enzyme stability and extend operational lifetime [8, 9]. Among various matrices, crystalline metal‐organic frameworks (MOFs) have emerged as promising scaffolds for immobilizing biomacromolecules owing to their high loading efficiency, tunable microenvironment, and strong protective capability [10]. So far, various MOFs have demonstrated efficient enzyme encapsulation and markedly improved stability [11, 12, 13]. For instance, MOF‐encapsulated enzymes can retain 80%–93% activity after 3 days at 4°C, compared to ∼30%–40% for free enzymes [14, 15, 16]. However, the intrinsically narrow pores of crystalline MOFs severely restrict substrate diffusion, resulting in low apparent activity (<20% of free enzymes) [17]. Consequently, there is an urgent need to develop new strategies to reconcile the inherent trade‐off between enzyme activity and stability in enzyme‐MOF systems.

To address this limitation, extensive efforts have been devoted to introducing structural defects into crystalline MOFs, thereby tailoring pore environments and improving substrate accessibility, which in turn enhances the catalytic performance of immobilized enzymes [18, 19, 20]. Nevertheless, defect‐engineered MOFs remain constrained by structural rigidity and non‐uniform defect distribution, which can induce enzyme leaching and limit mass transport [21, 22]. In contrast, amorphous MOFs (AMOFs), characterized by structural disorder and enhanced flexibility, alleviate diffusion limitations and improve substrate accessibility, thereby providing an adaptive microenvironment for enzyme encapsulation that preserves enzyme conformation and enhances catalytic activity [23, 24, 25]. Previous studies have demonstrated that enzymes immobilized in AMOFs exhibit superior catalytic activity compared to defect‐engineered MOFs [23, 26]. However, current strategies for enzyme immobilization using AMOFs typically rely on either the structural collapse of crystalline MOF [27] or external modulation strategies, including modulator‐induced disorder [28], defect accumulation [21], and kinetic control [29], to induce amorphization. These approaches often complicate synthesis, reduce reproducibility, and may not be broadly applicable across different enzymes. Notably, enzymes possess abundant functional groups that can coordinate with metal nodes, thereby participating in MOF growth and guiding framework assembly [30, 31]. This intrinsic regulatory capability provides a unique route to construct amorphous frameworks via enzyme‐mediated control, without the need for external modulators or structural collapse. However, the integration of enzyme‐mediated synthesis with amorphous MOF formation remains largely unexplored, highlighting the need to understand enzyme‐framework interactions for the rational design of enzyme‐mediated amorphous MOF systems with enhanced biocatalytic performance.

**SCHEME 1:**
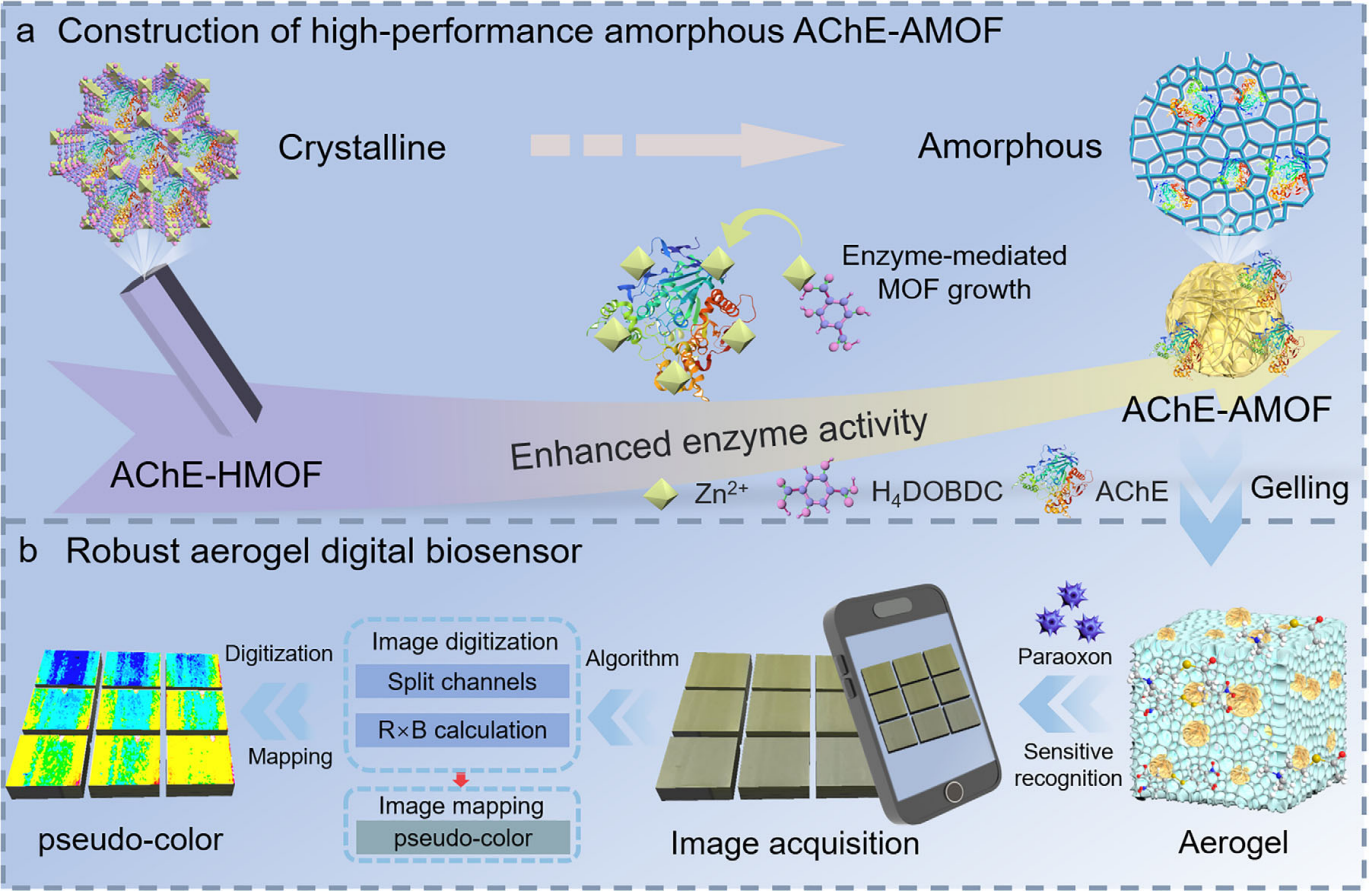
a) Schematic illustration of the enzyme‐mediated synthesis of high‐performance amorphous AChE‐AMOF. b) Robust AChE‐AMOF‐based aerogel digital biosensor for POC detection of OPs.

Herein, we report a robust aerogel digital biosensor (ADB) based on an enzyme‐mediated amorphous MOF (AChE‐AMOF) for on‐site detection of organophosphorus pesticides (OPs) (Scheme 1). Specifically, an enzyme‐mediated assembly strategy was employed to achieve the in situ encapsulation of AChE while transforming from a hexagonal prismatic AChE‐MOF (AChE‐HMOF) into an amorphous AChE‐AMOF nanostructure, thereby markedly enhancing the catalytic activity (Scheme 1a). This approach leverages the strong binding affinity between the enzyme and Zn^2+^, which facilitates enzyme‐mediated MOF nucleation and growth, and induces coordination defects within the framework. Notably, the amorphous AChE‐AMOF retains 82.3% of the catalytic activity of the free enzyme, corresponding to a 2.2‐fold higher activity than AChE‐HMOF, while still providing effective protection for the encapsulated enzyme. To further improve the stability and practical applicability, the AChE‐AMOF was embedded into a calcium(II)/alginate aerogel matrix to construct a portable ADB for POC applications (Scheme 1b). Benefiting from the dual protective effects of the AMOF framework and the aerogel matrix, the robust ADB retained over 98% enzymatic activity for 50 days, thereby significantly enhancing the stability of the biosensor for the monitoring of OPs. In addition, by digitizing the colorimetric signals using an image‐processing algorithm, the ADB enabled precise quantification of paraoxon, achieving a 10‐fold increase in detection sensitivity compared with a homogeneous liquid‐phase system. This study provides a practical approach to simultaneously enhancing both sensitivity and stability for on‐site pesticide detection, highlighting the potential of the ADB as a promising tool for advancing precision agriculture.

## 2 Results and Discussion

### 2.1 Synthesis and Structural Characterization of AChE‐AMOF

**FIGURE 1:**
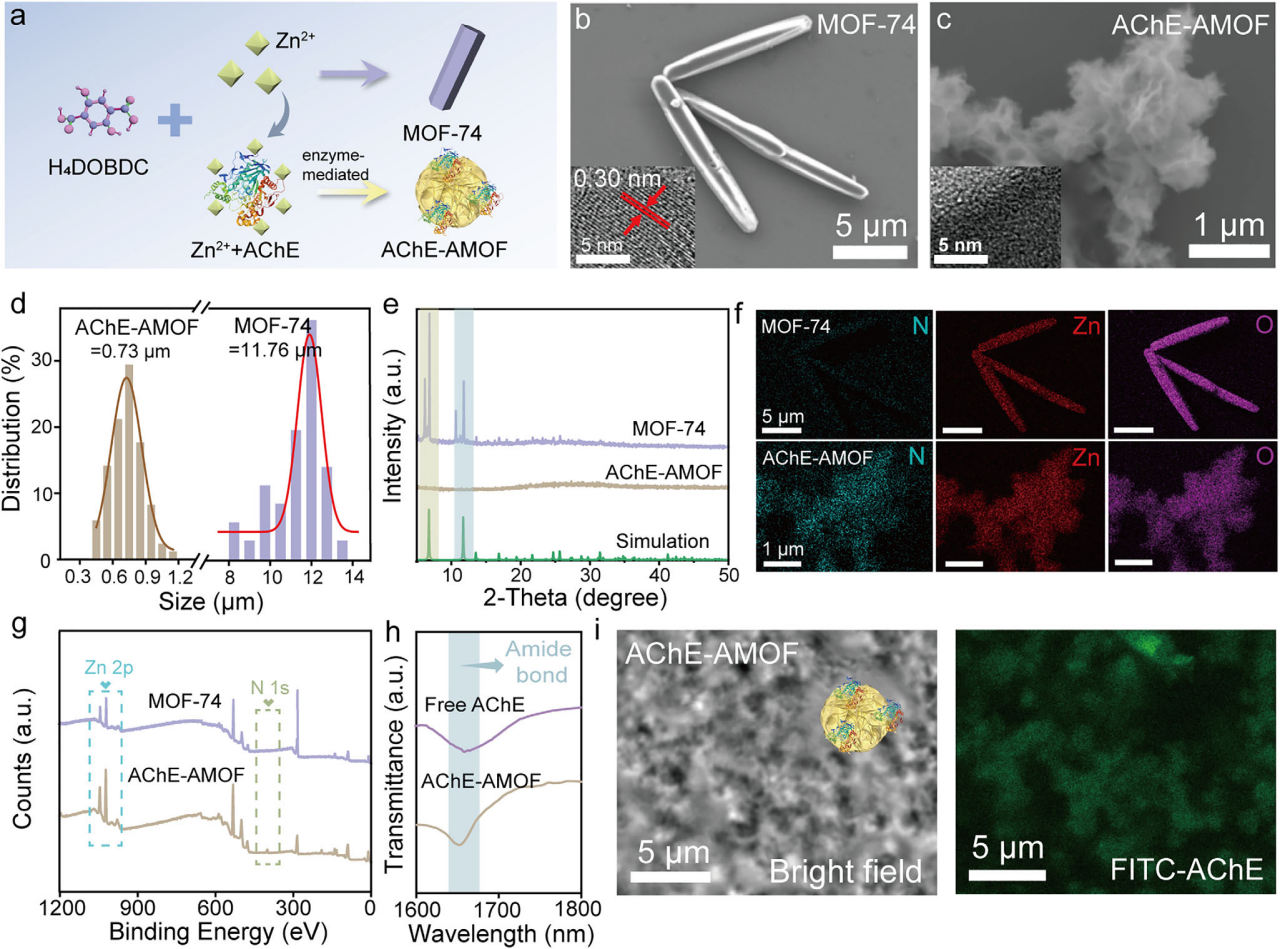
Synthesis and characterization of AChE‐AMOF. a) Schematic illustration of the enzyme‐mediated transformation of crystalline MOF‐74 into amorphous AChE‐AMOF. b) SEM image of MOF‐74 with an inset showing its HRTEM image, revealing a well‐defined crystalline lattice. c) SEM image of AChE‐AMOF with an inset showing its HRTEM image, indicating an amorphous structure without discernible lattice fringes. d) Size distribution comparison between MOF‐74 and AChE‐AMOF. e) XRD patterns of MOF‐74 and AChE‐AMOF, showing the transition from a crystalline to an amorphous phase after enzyme incorporation. f) EDS elemental mapping of AChE‐AMOF, demonstrating the homogeneous distribution of N, Zn, and O elements within the composite. g) XPS spectra of MOF‐74 and AChE‐AMOF, confirming the successful incorporation of AChE into the framework. h) FT‐IR spectra of free AChE and AChE‐AMOF, showing the characteristic amide band at 1653 cm^−1^ derived from the enzyme backbone. i) CLSM images of FITC‐labeled AChE within AChE‐AMOF, indicating uniform spatial distribution of the enzyme in the composite.

The effective resolution of stability challenges associated with biosensors is achieved through enzyme immobilization. Host MOF‐74 was specifically chosen for encapsulating enzymes owing to its aqueous phase preparation and coordination flexibility [32]. AChE, exhibiting strong interactions with metal ions, was employed as the model enzyme to demonstrate the feasibility of this protocol [33, 34]. During the enzyme‐mediated synthesis process, AChE was pre‐complexed with Zn(NO_3_)_2_·6H_2_O, thereby establishing a local enrichment of Zn^2+^ on the enzyme surface. After introducing the H_4_DOBDC ligand for 10 min, the amorphous AChE‐AMOF composite formed easily at room temperature through coordination between H_4_DOBDC and Zn(II) (Figure 1a). Scanning electron microscopy (SEM) analysis showed that pure MOF‐74 exhibits a relatively uniform hexagonal prismatic structure (average length = 11.76 µm) (Figure 1b,d). In contrast, the in situ immobilization of AChE in MOF‐74 exhibited a loose nanostructure similar to the core of a chrysanthemum (average size = 0.73 µm) (Figure 1c,d). High‐resolution transmission electron microscopy (HRTEM) revealed that the AChE‐AMOF composite adopts an amorphous structure lacking a discernible crystal lattice, in contrast to the well‐defined 0.30 nm lattice of crystalline MOF‐74 (Figure 1b,c). Consistently, the selected area electron diffraction (SAED) pattern of AChE‐AMOF exhibits broad diffuse halo‐like rings rather than discrete diffraction spots, indicating the absence of long‐range order, whereas crystalline MOF‐74 shows distinct diffraction features (Figure S1). X‐ray diffraction (XRD) analysis confirmed that pure MOF‐74 exhibited high crystallinity, consistent with previously reported patterns [32]. Following enzyme‐mediated transformation, the MOF transitioned from a crystalline structure to an amorphous architecture (no distinct 2θ peaks), indicating the regulatory effect of enzyme on MOF crystallization (Figure 1e). Energy‐dispersive X‐ray spectroscopy (EDS) elemental mapping showed a uniform distribution of N (from AChE), Zn, and O throughout AChE‐AMOF, indicating the presence of AChE within the composite (Figure 1f). X‐ray photoelectron spectroscopy (XPS) showed distinct Zn 2p and N 1s peaks, providing additional evidence of the effective incorporation of AChE within the AMOF framework (Figure 1g). Fourier‐transform infrared (FT‐IR) spectra further showed the appearance of an amide band at 1653 cm^−1^ in AChE‐AMOF, originating from the enzyme backbone and supporting the successful incorporation of AChE (Figure 1h). To assess the spatial accessibility of AChE within the composite, fluorescein isothiocyanate (FITC)‐labeled AChE was incorporated and visualized by confocal laser scanning microscopy (CLSM). The fluorescence images showed that AChE was homogeneously distributed throughout the AMOF matrix (Figure 1i). These results indicate that the introduction of AChE fundamentally alters the structure of MOF‐74, thereby enabling the in situ immobilization of the enzyme within the AChE‐AMOF composite.

### 2.2 Enzyme‐Mediated Structural Evolution of AChE‐AMOF

**FIGURE 2:**
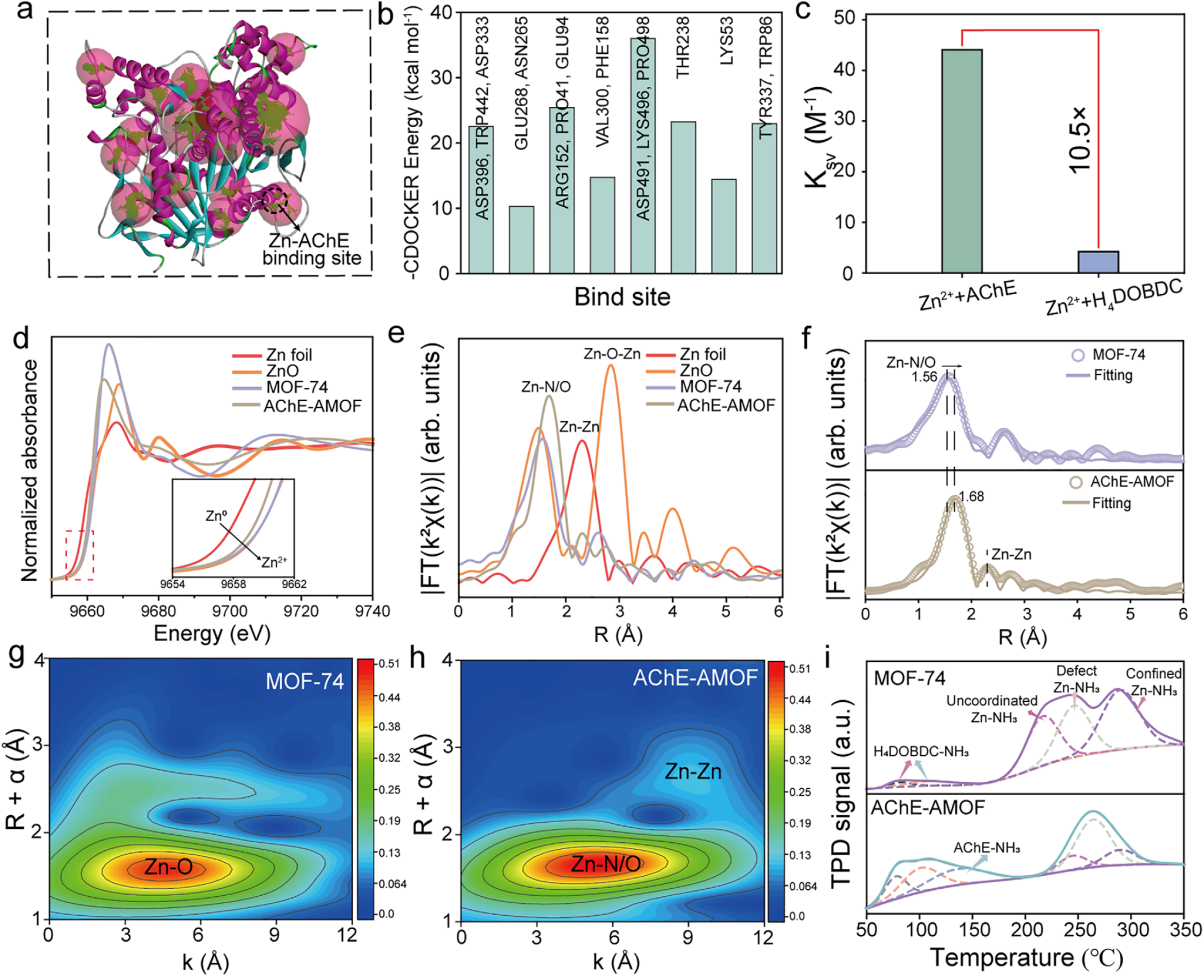
Enzyme‐mediated coordination reconstruction in AChE‐AMOF. a) Molecular docking results showing predicted Zn(II) binding sites on AChE (red spheres) (PDB: 1C2B). b) The ‐CDOCKER energy of key binding sites for AChE and Zn(II). c) Stern‐Volmer constants comparing Zn^2+^ interactions with AChE and H_4_DOBDC. d) Normalized Zn K‐edge XANES spectra of MOF‐74, AChE‐AMOF, Zn foil, and ZnO. e) k^2^‐weighted EXAFS Fourier transforms of Zn K‐edge for MOF‐74, AChE‐AMOF, Zn foil and ZnO. f) EXAFS fitting results showing local coordination environments of MOF‐74 and AChE‐AMOF. g, h) k^2^‐weighted EXAFS data subjected to wavelet transforms for g) MOF‐74 and h) AChE‐AMOF. i) NH_3_‐TPD profiles of MOF‐74 and AChE‐AMOF, indicating changes in surface active/defect sites induced by enzyme incorporation.

Evolution of morphological structure was unexpectedly observed upon the introduction of AChE, which differs from previous reports of in situ enzyme incorporation in other MOFs (such as ZIF‐8, ZIF‐90, and UIO‐66) [11, 12, 13]. To elucidate the formation mechanism of AChE‐AMOF composite, a systematic study of the interactions between AChE and MOF‐74 precursors (Zn^2+^ and H_4_DOBDC) is crucial. Molecular dynamics simulations revealed that Zn^2+^ preferentially forms Zn‐O/N coordination bonds with O‐ or N‐containing residues on AChE (e.g., Asp, Glu, Thr) with high binding energies (ΔE < ‐10 kcal·mol^−1^), indicating that coordination interactions are one of the main driving forces for Zn^2+^‐AChE binding (Figure 2a,b and Figure S2). Furthermore, zeta potential measurements showed that AChE effectively binds Zn^2+^ through electrostatic attraction, whereas it electrostatically repels H_4_DOBDC, resulting in minimal perturbation to the enzyme at the synthesis concentration (25 mm) (Figures S3 and S4). Therefore, Zn^2+^ can effectively associate with AChE through the combined contributions of coordination and electrostatic interactions. To quantify the interaction of Zn^2+^ with AChE and H_4_DOBDC, fluorescence quenching experiments were performed to determine the Stern‐Volmer constant (K_sv_). The K_sv_ value for Zn^2+^‐AChE was 10.5‐fold higher than that for Zn^2+^‐H_4_DOBDC, indicating that Zn^2+^ binds much more strongly to the enzyme surface residues (Figure 2c and Figures S5 and S6). Consistently, association constants (K_a_) derived from Scatchard analysis corroborated this trend, with the K_a_ of AChE toward Zn^2+^ being 7.4‐fold greater than that of H_4_DOBDC, demonstrating the markedly stronger binding affinity of AChE for Zn^2+^ (Figures S7 and S8). Taking these results into account, AChE effectively concentrates Zn^2+^ on its surface via strong enzyme‐metal interactions, disrupts the coordination of Zn‐H_4_DOBDC, and thereby promotes the formation of amorphous AChE‐AMOF structures.

Elucidating the electronic structure and coordination environment of Zn species in both MOF‐74 and AChE‐AMOF provides deeper insight into the enzyme‐mediated formation mechanism of AMOF. The Zn K‐edge X‐ray absorption near‐edge structure (XANES) spectra show that the absorption edge of MOF‐74 is nearly unchanged relative to the ZnO reference, indicating that Zn remains predominantly in the divalent state (Zn^2+^) and confirming that the metal framework retains its typical Zn‐H_4_DOBDC coordination environment (Figure 2d). In contrast, AChE‐AMOF exhibited an absorption edge at lower energy than that of MOF‐74, suggesting a modulation of the local electronic structure of Zn centers in AChE‐AMOF. This behavior may be associated with coordination interactions between Zn and functional groups of AChE, leading to a perturbed MOF‐74‐derived coordination environment and the formation of coordinatively unsaturated or distorted Zn sites. In the normalized k^2^‐weighted Fourier‐transform extended X‐ray absorption fine structure (FT‐EXAFS) spectra, pristine MOF‐74 shows a dominant Zn‐O peak at 1.56 Å (coordination number ≈ 4.0). In contrast, AChE‐AMOF exhibits a slightly broadened first‐shell peak at ∼1.68 Å, suggesting a modification in the local coordination environment of Zn centers after enzyme incorporation (Figure 2e,f and Table S1). Consistently, EXAFS wavelet transforms reveal enhanced intensity at 5.22 Å^−1^ corresponding to Zn‐N/O scattering and a new feature at 9.10 Å^−1^ associated with Zn─Zn scattering, further confirming that AChE incorporation alters both the local coordination and the extended structure of the MOF‐74 (Figure 2g,h). NH_3_ temperature‐programmed desorption (NH_3_‐TPD) analysis revealed pronounced differences in the desorption profiles of MOF‐74 and AChE‐AMOF. In the low‐temperature region (∼50°C–200°C), which reflects NH_3_ adsorption on non‐metal sites (uncoordinated H_4_DOBDC groups or AChE residues), MOF‐74 shows only a weak desorption feature. This originates from its highly ordered Zn‐H_4_DOBDC coordination environment, leaving very few uncoordinated ligand sites for NH_3_ binding (Figure 2i). In contrast, the incorporation of AChE disrupts the original Zn‐H_4_DOBDC coordination, generating a substantially larger number of uncoordinated H_4_DOBDC sites within the amorphous AChE‐AMOF framework. In the high‐temperature region (∼200°C–350°C), corresponding to typical NH_3_ adsorption on Zn sites, AChE‐AMOF displays only a small number of uncoordinated Zn sites (∼245°C) and confined Zn sites (∼289°C), while retaining a large number of defect Zn sites compared with MOF‐74 (Figure 2i).

Time‐resolved microscopy further reveals that this coordination modulation fundamentally alters the crystallization pathway. In the absence of AChE, MOF‐74 follows a typical nucleation‐growth process toward well‐defined hexagonal prisms (Figure S9). In contrast, strong Zn^2+^‐enzyme interactions in the presence of AChE induce localized ion enrichment and suppress ordered crystallization, leading to amorphous intermediates that assemble into nanoflower‐like architectures (Figure S10). Taken together, AChE directs the formation of AChE‐AMOF by preferentially binding Zn^2+^, thereby disrupting the Zn‐H_4_DOBDC coordination equilibrium and promoting the generation of amorphous intermediates. The subsequent aggregation of these intermediates drives the evolution toward nanoflower‐like architectures, establishing an enzyme‐mediated coordination modulation mechanism for AMOF formation.

**FIGURE 3:**
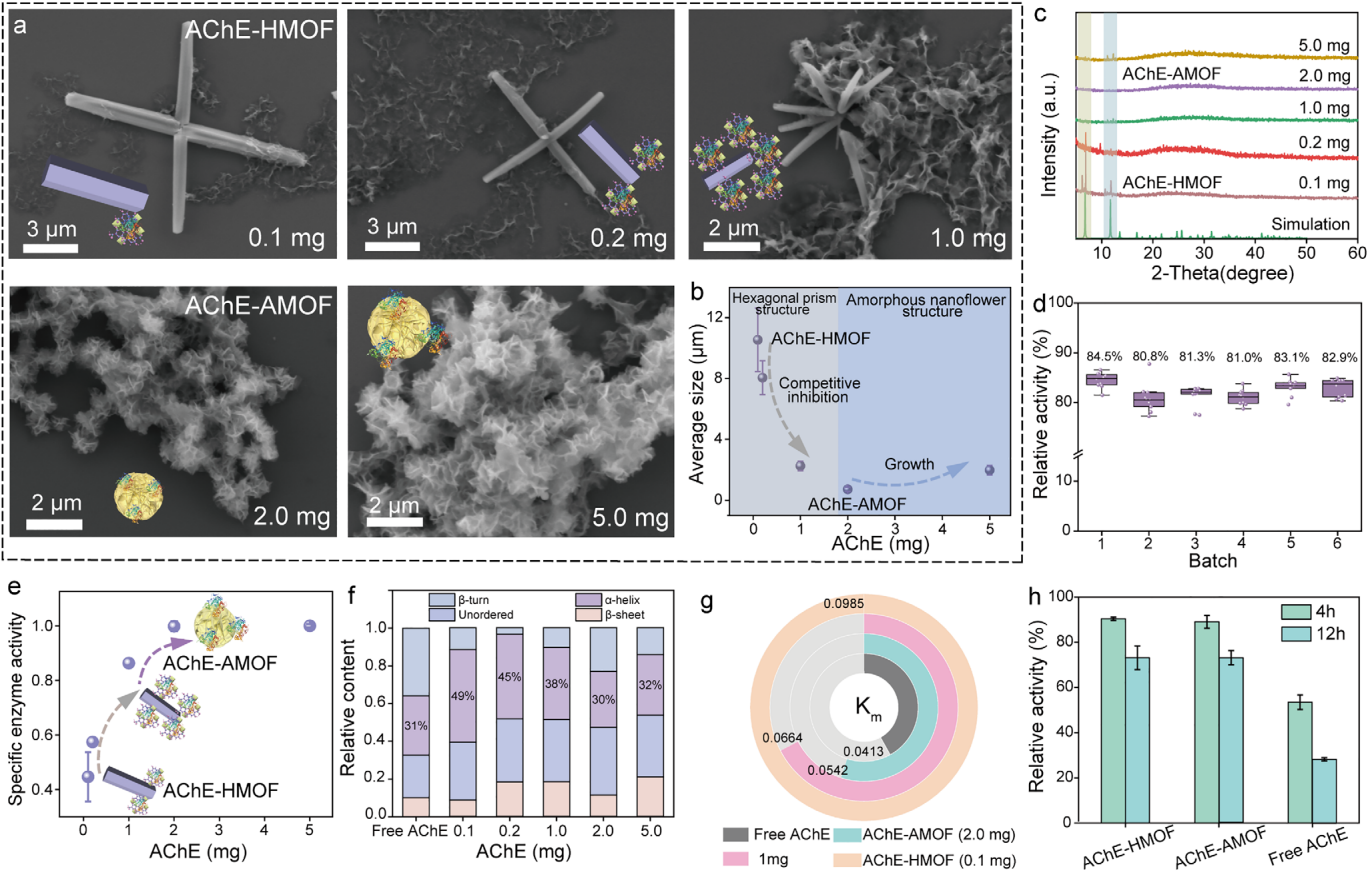
Structural evolution and performance regulation of AChE‐AMOF a) SEM images showing morphological evolution of AChE‐MOF synthesized with different amounts of AChE. b) Average size of AChE‐MOF at different amounts of AChE (Sizes correspond to the long‐axis dimension of the hexagonal prisms (AChE < 2.0 mg) and the diameter of the amorphous nanoflowers (AChE > 2.0 mg), respectively). c) XRD patterns of AChE‐MOF prepared with varying amounts of AChE, d) Batch‐to‐batch activity and reproducibility of AChE‐AMOF, showing activity distributions from six independent synthesis batches, each measured in ten independent replicates (box plot representation). e) The relative specific enzyme activity (enzyme activity divided by the amount of enzyme added) of AChE‐MOF prepared with different AChE amounts (AChE‐HMOF corresponds to 0.1 mg of AChE, while AChE‐AMOF corresponds to 2.0 mg of AChE). Data are presented as mean ± standard deviation (n = 3). f) Secondary structure analysis of free AChE and immobilized AChE in AChE‐MOF. g) Comparison of the K_m_ values of free AChE and AChE‐MOF synthesized with different AChE amounts. h) Proteolytic stability of AChE‐AMOF and AChE‐HMOF after incubation with trypsin for 4 and 24 h. Data are presented as mean ± standard deviation (n = 3).

To systematically investigate the influence of the enzyme on AChE‐MOF structure, composites were synthesized with varying amounts of AChE. At a low amount of added AChE (0.1 mg), SEM images revealed the formation of hexagonal prismatic AChE‐MOF with a well‐defined crystalline structure, which was defined as AChE‐HMOF (Figure 3a,c). As the AChE amount increased toward 1.0 mg, the size of these ordered prisms decreased from 10.54 to 2.26 µm, and the structures were progressively replaced by irregular aggregates (Figure 3a–c). These aggregates likely represent ternary complexes of Zn^2+^, AChE, and H_4_DOBDC, which may spontaneously phase‐separate in solution (Figure S11). Upon increasing the AChE amount to 2.0 mg, a clear phase transition occurred, with the hexagonal prismatic AChE‐HMOF structure completely disappearing and being replaced by an enzyme‐mediated amorphous AChE‐AMOF composite (Figure 3a,c). This transformation can be attributed to the excessive amount of enzyme disrupting the intrinsic nucleation and directional growth of the MOF framework. Further increasing the AChE amount to 5.0 mg, the nanoflower morphology gradually enlarged (Figure 3b), likely due to the high enzyme concentration further promoting aggregation of amorphous enzyme assemblies. Additionally, with the gradual increase in AChE concentration, the system progressively transitioned from a crystalline to an amorphous state (Figure 3c), meaning that AChE successfully mediated the morphology and crystal growth of MOF through competitive interactions.

Considering that alterations in crystal structure and morphology may affect the activity of immobilized enzymes, the enzymatic activity of AChE‐MOF prepared with different AChE amounts was evaluated by monitoring the chromogenic reaction of acetylthiocholine (ATCh) and 5,5'‐dithiobis (2‐nitrobenzoic acid) (DTNB) (Figures S12 and S13). To accurately and reproducibly determine the retained native enzymatic activity of AChE after immobilization in AMOF‐74, six independent batches of AChE‐AMOF were synthesized. Each batch was subjected to ten independent activity measurements to account for variations arising from sample preparation and measurement procedures (Figure 3d and Figure S14). As shown in Figure 3d, different batches of AChE‐AMOF exhibited consistent and high relative activity (%) to free AChE, ranging from 80.8% to 84.5%, with a mean value of 82.3%. Impressively, despite comparable protein loading efficiency (%), AChE‐AMOF exhibited a 2.2‐fold higher specific enzyme activity (defined as enzyme activity normalized to the amount of enzyme added) compared with AChE immobilized in crystalline hexagonal prismatic AChE‐HMOF (Figure 3e and Figure S15). Furthermore, increasing the AChE amount from 2.0 to 5.0 mg does not significantly affect its specific activity, likely because both samples possess a similar high‐performance amorphous structure. To elucidate the origin of the enhanced catalytic activity, we first evaluated the effects of MOF precursors on AChE activity. Both Zn^2+^ and H_4_DOBDC at the synthesis concentration (25 mM) showed negligible influence, indicating that the enhanced activity of AChE‐AMOF does not arise from precursor‐enzyme interactions (Figure S16). The α‐helical content of the enzyme is a key structural parameter that governs its catalytic performance. Gaussian fitting analysis of the secondary structure showed that the α‐helical content of AChE‐AMOF was comparable to that of free AChE, indicating that the native secondary structure was largely preserved and providing structural evidence for the high enzymatic activity observed (Figure 3f). Steady‐state kinetic analysis based on the Michaelis‐Menten model showed that AChE‐AMOF exhibited a K_m_ of 0.0542 mmol L^−1^, comparable to that of free AChE (0.0413 mmol L^−1^) and significantly lower than that of AChE‐HMOF (K_m_ = 0.0985 mmol L^−1^), demonstrating that the AChE‐AMOF preserves high substrate affinity (Figure 3g and Figure S17). Nitrogen adsorption measurements revealed that the average pore size of MOF‐74 increased from 9.8 nm to 21.8 nm as the amount of AChE was increased from 0 to 2.0 mg (Table S2), thereby enhancing substrate transport and improving enzymatic activity. Consequently, the enhanced activity is primarily attributed to the amorphous structure, which modulates the physical and chemical microenvironment of immobilized enzyme, improving substrate accessibility, enlarging pore size, and preserving the native enzyme structure, thereby promoting catalytic performance. An optimal enzyme immobilization strategy should preserve enzyme stability while maximizing catalytic activity. To evaluate the protective effects of the crystalline and amorphous AChE‐MOF structures on AChE activity, AChE‐HMOF, AChE‐AMOF, and free AChE samples were incubated with trypsin for 4 and 24 h, after which their enzymatic activity was measured. The results indicate that both the amorphous AMOF and crystalline HMOF structures effectively protect the immobilized enzyme, exhibiting comparable protection against proteolytic degradation (Figure 3h).

The generalizability of this method was further validated by encapsulating horseradish peroxidase (HRP) and glucose oxidase (GOX) within MOF‐74 using the same approach, yielding amorphous enzyme–AMOF structures similar to AChE (Figures S18 and S19). Notably, benefiting from the amorphous structure, the enzymatic activities of GOX‐AMOF and P‐HRP‐AMOF were enhanced by 1.43‐fold and 2.29‐fold, respectively, compared with their crystalline counterparts (Figure S18). These results demonstrate that the enzyme‐mediated strategy is broadly applicable to different enzymes and enables simultaneous structural modulation and activity enhancement in enzyme‐MOF systems.

### 2.3 Construction of AChE‐AMOF‐Based Aerogel Digital Biosensors (ADB)

**FIGURE 4:**
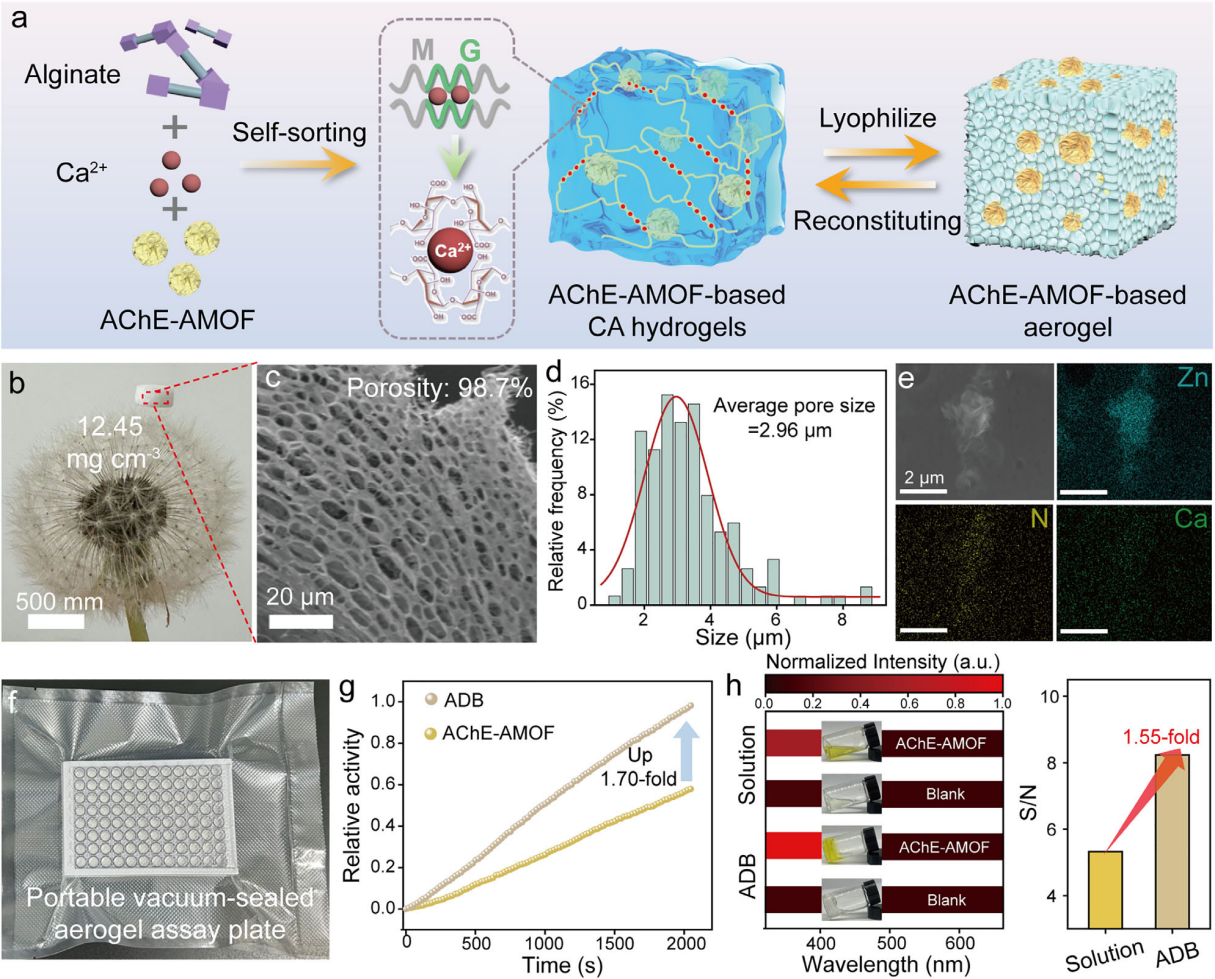
Construction of aerogel digital biosensor (ADB). a) Schematic illustration of ADB fabrication based on AChE‐AMOF incorporated Ca^2+^/alginate hydrogel and freeze‐drying process. b) Photograph of the super‐light freeze‐dried aerogel on top of a dandelion. c) SEM image of AChE‐AMOF‐aerogel. d) Relative frequency (%) distribution of AChE‐AMOF aerogel pore sizes. e) Elemental mapping of N, Zn, and Ca, confirming uniform distribution of AChE‐AMOF within the aerogel matrix. f) A photograph of the ADB obtained through vacuum‐sealed freeze‐drying in a commercial 96‐well plate. g) Comparison of relative enzymatic activity between AChE‐AMOF in the liquid phase and ADB system. h) Comparison of colorimetric signal intensity between the liquid‐phase sensing platform and the aerogel sensing platform.

To enable POC testing, the AChE‐AMOF was incorporated into engineered 3D Ca^2+^/alginate (CA) hydrogel carriers for the preparation of the ADB. In this process, Ca^2+^ mediated the self‐assembly of alginate chains into a colorless and transparent hydrogel network, providing an optimal substrate for colorimetric sensing (Figure 4a). This was confirmed by its transmission variation of less than 10% within the 450–700 nm range, which ensures minimal optical interference and accurate color signal detection (Figure S20). The AChE‐AMOF‐based CA hydrogels were subsequently freeze‐dried to obtain lightweight aerogels with an average density of 12.45 mg cm^−3^, lower than that of previously reported aerogel biosensors [35, 36] (Figure 4a,b and Figure S21). SEM images reveal that the ADB exhibits an interconnected 3D fibrous network formed by entangled fibers and junctions, forming a porous aerogel with an average pore size of 2.96 µm and a high porosity of 98.7% (Figure 4c,d and Table S3). EDS mapping confirms the co‐localization of N, Zn, and Ca signals, demonstrating the successful encapsulation of AChE‐AMOF within the aerogel network (Figure 4e). To evaluate the rapid activation performance of the ADB upon contact with water, the water absorption and expansion behavior of the ADB were monitored at various time intervals. Upon contact with water, the ADB rapidly absorbed liquid and released entrapped air bubbles, indicating immediate network swelling and pore activation. Remarkably, 2.5 mg of ADB rapidly absorbed approximately 250 µL of water (100 times its own mass) and reconstituted into a colorless, transparent hydrogel within 90 s (Figure S22). Benefiting from its lightweight, highly porous architecture, the ADB rapidly absorbs water, promoting enzyme reactivation and substrate diffusion [37], which in turn markedly shortens detection preparation time and enables timely POC applications. To expand the detection throughput and enable portable operation, a commercial 96‐well plate was used to fabricate a vacuum‐sealed aerogel assay platform (Figure 4f), which ensures low per‐sample cost (≈0.2 CNY) and facile fabrication, thereby enabling rapid, on‐demand deployment. Notably, the reaction rate significantly increased (1.70‐fold) within the aerogel systems in comparison to a homogeneous liquid‐phase system (Figure 4g). The intensity response of the –SH group in water was consistent with that in the aerogel, indicating that the gel does not interfere with the thiocholine/DTNB colorimetric reaction (Figure S23). Therefore, theincreased reaction rate can be attributed to the spatial confinement effect of the gel for boosting the enzyme‐substrate contact [38]. A visible colorimetric change with high contrast to the background can be observed in the ADB platform, confirming that the obtained signal‐to‐noise ratio (S/N) is significantly improved 1.55‐fold in the ADB compared to the homogeneous liquid‐phase system (Figure 4h). Taken together, the developed ADB not only provides a structurally stable and portable POC sensing platform, but also significantly enhances catalytic efficiency and signal‐to‐noise ratio, thereby supporting the development of an efficient and robust POC biosensor.

**FIGURE 5:**
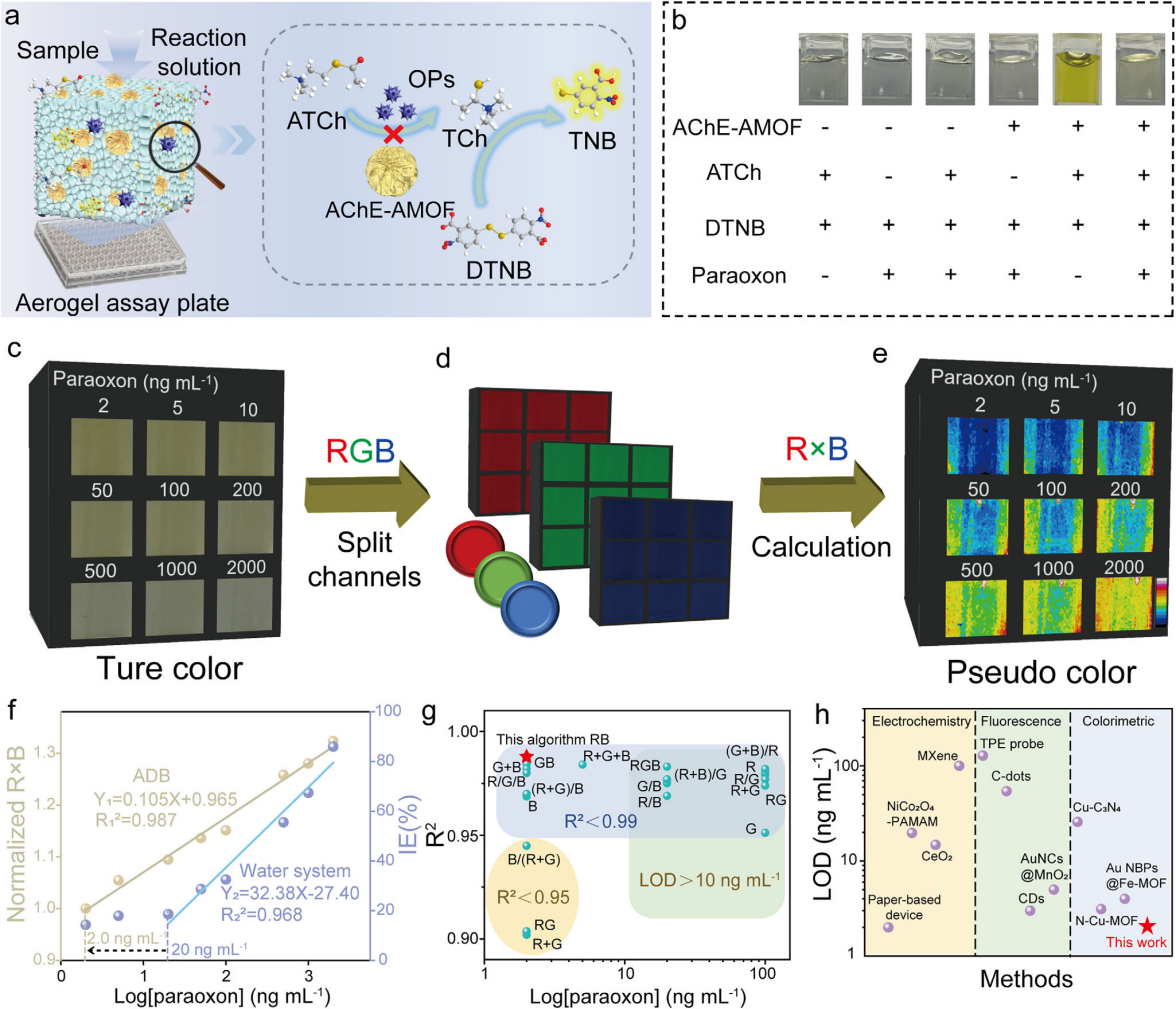
Sensing performance of the ADB platform. a) Schematic illustration of the ADB‐based colorimetric sensing mechanism for paraoxon detection. b) Feasibility of paraoxon detection based on AChE inhibition in the ADB system. c) True color images of ADB at different paraoxon concentrations. d) Corresponding R, G, and B channel images of the ADB under different paraoxon concentrations. e) Pseudo color R×B response images calculated from the pixel arrays of the R and B channels. f) Linear fitting of signal response versus paraoxon concentration for the aerogel (ADB, R×B parameter) and liquid‐phase system (inhibition efficiency, IE%). g) Effect of different digital fitting methods on the LOD and linearity for paraoxon quantification. h) Comparison of the detection limits of this work with those of previously reported methods.

In precision agriculture, the rapid and sensitive detection of pesticides not only ensures the safety and quality of agricultural products but also enables timely adjustment of pesticide inputs, which played a crucial role in the smooth operation of monitoring systems. Paraoxon, a representative organophosphorus pesticide, was chosen as the model analyte due to its strong toxicity and risk of food contamination [39, 40]. The sensing abilities of ADB, which relied on AChE catalyzing the hydrolysis of substrate and paraoxon‐induced enzyme inhibition, were investigated via collecting colorimetric signals (Figure 5a). The results demonstrated that paraoxon efficiently inhibited AChE‐AMOF activity, leading to a pronounced colorimetric response in the ADB, thereby confirming its potential for sensitive pesticide detection (Figure 5b). To quickly analyze the readout signals, true‐color images were captured using a smartphone under a fixed arrangement of the device, sample, and light source (Figure 5c and Figure S24). The images were then digitized to separate the color signals of the photos into three primary color codes (RGB: red, green, blue) and converted them into digital information. Following exposure to paraoxon, the digital color responses of ADB changed proportionally with the analyte concentration (Figure 5d and Figure S25). In order to clearly present the results, the color signals of the samples were digitized to generate an interpretable pseudo‐color difference map (Figure 5e). Subsequently, the correlation between paraoxon concentration and various colorimetric fitting algorithms was then evaluated, showing that the R×B parameter provided a suitable linear relationship (Figure 5f,g). The fitting results showed that the R×B signal exhibited a linear relationship with paraoxon concentration over the range of 2–2000 ng mL^−1^, described by the equation: Y = 0.105 Log[paraoxon]+0.965 (Figure 5f). Notably, the image digitization algorithm reduced the limit of detection (LOD) to 2.0 ng mL^−1^, representing a 10‐fold improvement compared with conventional homogeneous solution methods. This approach also surpassed other color‐processing methods as well as previously reported pesticide sensors, highlighting the effectiveness of the R×B parameter to ADB for pesticide detection (Figure 5g,h and Table S4). Such excellent detection performance can be ascribed to the following characteristics: i) The enzyme‐mediated amorphous AChE‐AMOF architecture introduces coordination defects that enlarge the MOF pores, thereby facilitating substrate conversion and enhancing catalytic activity ii) the highly porous aerogel matrix‐derived confined microenvironment promotes enzyme‐substrate interactions, leading to an accelerated reaction rate and improved signal‐to‐noise ratio iii) By employing an image digitization algorithm to convert color gamut, ADB can not only enhance the accuracy and sensitivity of the corresponding color via amplifying the response signal but also directly acquire results without the need for a precision instrument.

### 2.4 Practical Robustness of ADB for POC Applications

**FIGURE 6:**
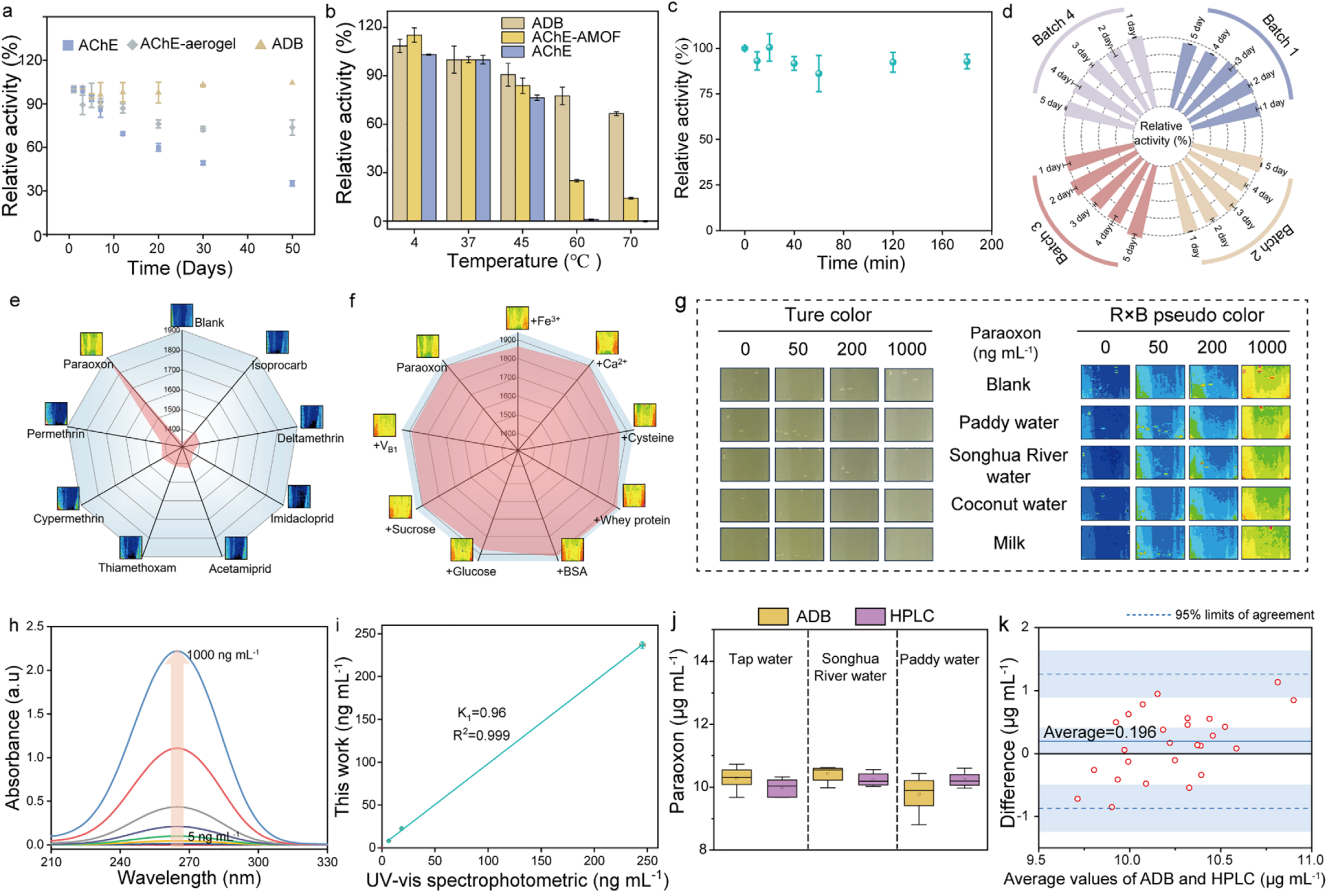
Analytical performance and real‐sample validation of ADB a) Storage stability of free AChE, AChE‐aerogel, and ADB over time. b) Temperature stability of free AChE, AChE‐AMOF, and ADB. c) Stability of the ADB under UV irradiation (365 nm, 12 W). d) Batch‐to‐batch reproducibility and temporal stability of ADB over five consecutive days for four independently prepared batches. Data are presented as mean ± standard deviation (n = 3). e) Selectivity of ADB toward paraoxon in the presence of other pesticides. f) Anti‐interference performance of ADB in the presence of common substances. g) True color image and R×B pseudo color of ADB spiked with real samples. h) UV–vis absorption spectra of paraoxon at different concentrations (5–1000 ng mL^−1^), showing its characteristic absorption at 265 nm. i) Correlation between the ADB‐based platform and conventional UV–vis spectrophotometric method for paraoxon quantification. j) The box‐whisker plot of HPLC and ADB. Data pertain to twelve parallel experiments for each water sample (n = 12). k) Bland‐Altman analysis was used to evaluate the agreement between this work and HPLC.

The long‐term stability is a crucial determinant of the practical reliability of biosensors. Accordingly, the stability of the ADB was systematically assessed during extended storage. Remarkably, the biosensor maintained over 98% of its initial activity after 50 days, which is substantially higher than that of AChE‐aerogel controls (74% retained) and free AChE (35% retained), underscoring its exceptional stability and outperforming previously reported enzyme‐based biosensors (Figure 6a) [41, 42]. In addition, thermal stability tests further highlighted the robustness of the ADB. Free AChE rapidly lost nearly all catalytic activity at 60°C, whereas AChE‐AMOF preserved approximately 25% of its initial activity under the same conditions. By contrast, the ADB exhibited markedly enhanced thermal resistance, retaining 67% of its original activity even at 70°C (Figure 6b). The ADB also exhibited excellent photostability, with no noticeable change in enzymatic activity after 30 h of ambient light exposure (Figure S26). Even under strong UV irradiation (365 nm, 12 W) for 180 min, the activity remained above 90% of its initial value, demonstrating robust stability under both ambient light and UV exposure (Figure 6c). Mechanical robustness is an important parameter for evaluating practical applicability. Compression tests showed that strains from 0 to 90% had negligible influence on enzymatic activity, with variations within 10% (Figures S27 and S28), indicating that the ADB maintained stable sensing performance under substantial mechanical deformation. The enhanced stability of the ADB arises from the synergistic protection provided by the AMOF‐supported enzyme architecture and the aerogel matrix. The porous AMOF structure provides a relatively stable microenvironment that shields the enzyme from environmental stress, while the aerogel network offers a buffered matrix that mitigates thermal and storage‐induced denaturation. Therefore, the ADB offered high stability, mitigating the effects of high temperatures and storage concerns.

Reliable detection in real‐world environments requires high batch‐to‐batch reproducibility, selectivity, and strong anti‐interference capability. To evaluate reproducibility, four independently prepared batches were tested over five consecutive days. All batches exhibited highly consistent activity signals, demonstrating excellent reproducibility and supporting the suitability of the ADB for large‐scale manufacturing (Figure 6d). The selectivity of the ADB toward paraoxon was evaluated using various common pesticides as negative controls. As depicted in Figure 6e, the R×B values of high concentration common pesticides (e.g., isoprocarb, deltamethrin, imidacloprid, acetamiprid, thiamethoxam, cyhalothrin, and permethrin) (5.0 µg mL^−1^) closely resembled that of the blank group and were significantly lower than the response to paraoxon (1.0 µg mL^−1^). These results indicated the high specificity of ADB in detecting paraoxon. To emulate the real environments, common substances (proteins, amino acids, and ions) were tested for interference resistance. These common substances did not cause significant changes in R×B (within 10%, Figure 6f). The exceptional selectivity and anti‐interference performance of the ADB primarily stems from the AChE's intrinsic specificity toward OPs. Furthermore, the presence of paraoxon in food and environmental samples (including Chinese cabbage, fruit samples, milk, paddy water, Songhua River water, and coconut water) was detected to assess practicality and reliability (Figure 6g and Table S5). The recovery rates ranged from 95.19% to 105.16%, with relative standard deviations (n = 3) below 3.46%, indicating good accuracy in complex matrices. The reliability of the ADB was validated using UV–vis spectrophotometry as a benchmark (Figure 6h and Figure S29). The two methods showed excellent agreement, with a linear regression slope of 0.96 (R^2^ = 0.999), and the proposed method maintained high accuracy in the presence of typical interferents (NaCl and glucose), with regression slopes close to unity (0.97 and 1.00, respectively) (Figure 6i and Figure S30). The recoveries obtained with the ADB were further validated against High‐Performance Liquid Chromatography (HPLC), with box plots from twelve parallel measurements for each water sample (n = 12) revealing a high degree of consistency between the two methods (Figure 6j). Bland‐Altman analysis showed a mean difference of 0.196 µg·mL^−1^, corresponding to 1.91% of the average measurement (Figure 6k). Taken together, the AChE‐AMOF‐based ADB demonstrated satisfactory accuracy in complex matrices. ADB exhibited excellent sensitivity and stability, facilitating on‐site detection of pesticide residues in agricultural products by analyzing pesticide characteristics, thereby promoting the development of sustainable agriculture.

## 3 Conclusion

In summary, a robust AChE‐AMOF‐based ADB was developed for on‐site and sensitive detection of pesticide residues. The amorphous AChE‐AMOF was constructed through enzyme‐mediated MOF self‐assembly. In this process, AChE induces Zn^2+^ aggregation, promoting nucleation and growth of the MOF, while perturbing the dense MOF‐74 coordination network and generating nanoflower‐like porous structures. These structural features provide a favorable microenvironment for enzyme immobilization, thereby enhancing the overall catalytic performance. Impressively, AChE‐AMOF retains up to 82.3% of thefree enzyme activity, corresponding to a 2.2‐fold increase in catalytic activity compared with AChE loaded in crystalline hexagonal‐prism MOF structures. This superior activity can be attributed to the coordination defects and enlarged pore size of AChE‐AMOF, which facilitate substrate diffusion and enhance signal transduction. On the basis of these promising features, we encapsulated AChE‐AMOF within specially designed Ca(II)/alginate hydrogels to develop AChE‐AMOF‐based ADB. Benefiting from the combined protective effects of the AMOF framework and the aerogel matrix, the robust ADB exhibits substantially enhanced stability, maintaining over 98% of its enzymatic activity for 50 days. Furthermore, the biosensor's colorimetric responses were digitized and further refined using a signal‐optimization algorithm to improve practical on‐site applicability, enabling the ADB to achieve a LOD as low as 2.0 ng·mL^−1^. This work not only provides an effective strategy for designing robust and sensitive enzyme‐AMOF nanoarchitectures but also demonstrates their practical potential for enzyme‐based POC biosensor applications.

## 4 Experimental Section

### 4.1 Synthesis of AChE‐AMOF

A 2 mg portion of AChE was mixed with Zn(NO_3_)_2_·6H_2_O (14.994 mg) and stirred for 5 min to allow thorough Zn^2+^ accumulation on the enzyme surface. H_4_DOBDC (10 mg) was then added, all dissolved in 8.0 mL of 10 mm Tris‐HCl buffer (pH 8.0). The mixture was stirred at 25 °C for 10 min. The resulting precipitates were collected by centrifugation at 7000 rpm for 10 min, washed thoroughly with ultrapure water, and subsequently redispersed for further use.

### 4.2 Preparation of AChE‐AMOF‐Based Aerogel

Sodium alginate (SA) was first dissolved completely in water and then transferred to a 96‐well plate (20.0 mg mL^−1^, 200 µL per well). Subsequently, 25 µL of AChE‐AMOF dispersion was added to the viscous SA solution and mixed thoroughly. Afterward, 25 µL of CaCl_2_ solution (5.0 mg mL^−1^) was introduced into the homogeneous AChE‐AMOF‐SA mixture to trigger gelation, yielding AChE‐AMOF‐based CA hydrogels. The hydrogels were then freeze‐dried to obtain the AChE‐AMOF‐based aerogel.

### 4.3 Real Sample Detection

Three actual samples of Songhua River water, paddy water, coconut water, and milk were used to test the feasibility of the colorimetric system. The samples were filtered through a 0.22 mm membrane to remove the precipitated substances. The filtered milk samples were diluted 50 times to attenuate the color interference with the system. The treated samples were spiked with 50, 200^,^ and 1000 ng mL^−1^ of paraoxon for paraoxon detection. For fruit and vegetable samples, pretreatment was performed according to the Chinese National Standard GB/T 5009.199‐2003. A phosphate buffer (pH 8.0) was prepared by dissolving 11.9 g of anhydrous dibasic potassium phosphate and 3.2 g of monobasic potassium phosphate in 1000 mL of distilled water. Representative samples were washed to remove surface contaminants and cut into ∼1 cm^2^ pieces. After spiking with paraoxon at the same concentrations (50, 200, and 1000 ng mL^−1^), 5 mL of the buffer was added. The mixtures were shaken for 10 min, and the resulting extracts were collected for analysis.

## Author Contributions

**Changshun Su**: conceptualization, writing – original draft, visualization, data curation, software, methodology. **Xiangyu Zhai**: validation, formal analysis, supervision. **Houru Li**: formal analysis, supervision. **Yijie Wang**: visualization, investigation. **Hongxia Li**: writing – review and editing, writing – original draft, conceptualization, methodology, funding acquisition. **Yueyao Jiang**: data curation, writing – original draft, writing – review and editing, funding acquisition. **Geyu Lu**: funding acquisition, formal analysis. **Xu Yan**: writing – review and editing, methodology, funding acquisition, supervision.

## Conflicts of Interest

The authors declare no conflict of interest.

## Supporting information




**Supporting File 1**: advs75997‐sup‐0001‐SuppMat.docx.

## Data Availability

The data that support the findings of this study are available from the corresponding author upon reasonable request.
