# Supplementary material for: Robust Amorphous MOF‐Based Aerogels Digital Biosensor for Sensitive Detection of Organophosphate Pesticides
Source: Adv Sci (Weinh). 2026 Jun 9:e75997. Online ahead of print. doi: 10.1002/advs.75997 (PMC13336996; doi:10.1002/advs.75997)
Supplement: Supplementary file 1 — Supporting File 1: advs75997‐sup‐0001‐SuppMat.docx. [file ADVS-9999-e75997-s001.docx]

**Supporting information**

**Robust Amorphous MOF-Based Aerogels Digital Biosensor for Sensitive Detection of Organophosphate Pesticides**

Changshun Su^1^, Xiangyu Zhai^1^, Houru Li^1^, Yijie Wang^2^, Hongxia Li^1,^*, Yueyao Jiang^3,^*, Geyu Lu^2^, Xu Yan^2,^*

*1 Department of Food Quality and Safety, College of Food Science and Engineering, Jilin University, Changchun 130062, China*

*2 State Key Laboratory on Integrated Optoelectronics, Key Laboratory of Advanced Gas Sensors of Jilin Province, College of Electronic Science & Engineering, Jilin University, Changchun 130012, P. R. China*

*3. Department of Pharmacy, China–Japan Union Hospital of Jilin University, Changchun, Jilin Province, 130033, China.*

** Corresponding author*

*Email:* *hxiali@jlu.edu.cn*

*yanx@jlu.edu.cn*

*jyy0705@jlu.edu.cn*

**Chemicals and materials**

Zinc nitrate [Zn(NO_3_)_2_•6H_2_O] was purchased from Aladdin Chemical Reagent Co., Ltd. (Shanghai, China). 2,5-Dihydroxyterephthalic acid (H_4_DOBDC), acetylthiocholine (ATCh), acetylcholinesterase (AChE), Tris-HCl, and 5, 5'-Dithiobis-(2-nitrobenzoic acid) (DTNB) were all purchased from Shanghai Macklin Biochemical Technology Co., Ltd. Bovine serum albumin (BSA) was obtained from Ryon Biological Technology Co. Ltd. (Shanghai, China). CaCl_2_ were got from Aladdin Reagent Co. Ltd (Shanghai, China). Cysteine (Cys), Glucose (Glu), Whey protein, Ovalbumin (OVA), Trypsin (Try), and proteinase K were obtained from Beijing Dingguo Changsheng Biotechnology Co., Ltd. Paraoxon, isoprocard, deltamethin, imidacloprid, acetamiprid, thiamethoxam, cyhalothrinnd and other pesticide samples were obtained from Tianjin Zhongyi technology Co., Ltd.

**Apparatus**

The microstructures are recorded using JEM-2100 transmission electron microscope (TEM) and JEM-7500 scanning electron microscope (SEM). X-ray photoelectron spectroscopy (XPS) measurement is performed using an American Thermo Fisher 250XI photoelectron spectrometer. The phase and crystalline information of the samples is examined by XRD (Rigaku D/MAX-2550, Cu-Kα, λ = 1.5418 Å). Fourier Transform Infrared (FT-IR) spectra were recorded on a Hitachi F-7100 spectrometer. The absorption spectrum was measured by Shimadzu UV-270001 ultraviolet-visible spectrophotometer. The absorbance values were recorded on a Thermo Fisher (Shanghai) multifunctional microplate reader Varioskan LUX.

**Preparation of MOF-74**

Typically, zinc nitrate (14.992 mg) was dissolved in 4 mL of Tris-HCl (50 mM, pH = 8). 2,5-Dihydroxyterephthalic acid (H_4_DOBDC, 10 mg) was dissolved in 4 mL of Tris-HCl (50 mM, pH = 8). The mixture was stirred at room temperature for 10 min. Finally, the precipitate was collected by centrifugation at 7000 rpm for 10 min, washed with ultrapure water and dispersed in ultrapure water.

**Specific enzyme activity evaluation**

Aliquots (25 μL) of free AChE, AChE-HMOF, and AChE-AMOF were each treated with 25 μL of H₂O, followed by the addition of 50 μL of PBS (10 mM), 50 μL of acetylthiocholine (ATCh, 10 mM), and 50 μL of DTNB (0.3 mg mL^-1^). The mixtures were thoroughly homogenized and incubated at 37 °C for 30 min. The absorbance was measured at 412 nm. Specific enzyme activity was defined as enzyme activity normalized to the amount of enzyme added. Data are presented as mean ± standard deviation (n = 3).

**Stability evaluation**

25 μL of free AChE, AChE-HMOF and AChE-AMOF was treated with 25 μL of trypsin for 4h or 12h, respectively. Subsequently, 50 μL of PBS (10 mM), 50 μL of ATCh (10 mM), 50 μL of water and 50 μL of DTNB (0.3 mg mL^-1^) were added and mixed homogeneously at 37°C for 30 min. The absorbance was measured at 412 nm, Data are presented as mean ± standard deviation (n = 3).

**Calculation of Inhibition Efficiency (IE)**

The IE value is calculated using the following Equation:

$$IE\left( \% \right)=\frac{(A-A_{1})}{(A-A_{0})}\times100\%$$

where A_0_ is the baseline intensity of the colorimetric sensing platform, A is the absorption intensity of the AChE-AMOF colorimetric sensing platform, and A_1_ is the absorption intensity of the system after adding different concentrations of paraoxon.

**Enzyme kinetic studies**

The kinetic behavior of AChE-MOF immobilized acetylcholine ester was investigated and the performance of immobilized acetylcholine ester was evaluated. 25 μL of catalyst, containing AChE, AChE-AMOF and AChE-HMOF, respectively, were added to a mixture containing 50 μL of PBS (10 mM, pH = 7.0), 50 μL of water, 50 μL of ATCh at different concentrations, and 50 μL of DTNB (0.3 mg mL^-1^) in a mixed solution. The change in absorbance with time was immediately detected and the kinetic curve was plotted. The kinetic parameters (K_m_ and V_max_) are calculated by the Michaelis-Menten equation:

1/V = (K_m_ / V_max_) (1/[S] + 1/V_max_)

where [S] stands for the concentration of substrate, V stands for the reaction rate at this concentration of the substrate.

**The sensing mechanism**

In this work, acetylcholinesterase (AChE) could catalyze hydrolysis acetylthiocholine to produce TCh, which regulates the absorbance intensity. The reaction that is catalyzed by AChE i:


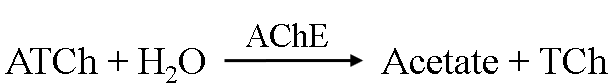


Specifically, AChE catalysis occurs when the anionic binding site of the catalytic coordinated triad (histidine, serine, and aspartic acid) attracts the positively charged thiocholine. The serine hydroxyl group attacks and cleaves the ester after its deprotonation by a neighboring histidine group in the triad. Thus, the TCh could trigger the decomposition of DTNB to TNB with a characteristic absorption peak around 412 nm, accompanying a distinguishable color change.


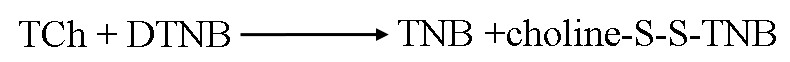


Paraoxon could covalently bind the triad's anionic binding site of AChE to form a phosphorylated enzyme, blocking the activity of the enzyme and inducing the change of absorbance intensity of the system.

**Binding affinities of Zn²⁺ with AChE and H_4_DOBDC via fluorescence spectroscopy**

To gain a deeper understanding of the interaction forces among Zn^2+^, H_4_DOBDC, and AChE, the inherent fluorescence properties of AChE and H_4_DOBDC were employed to investigate their binding affinities with Zn^2+^. Specifically, different concentrations of Zn²⁺ (0-50 nM) were mixed with 0.5 mg mL⁻¹ of AChE and allowed to react for 10 minutes at 25°C. The resulting solutions were then analyzed using a Hitachi F-7100 spectrometer (Ex = 282 nm) to obtain the fluorescence spectra. Similarly, the interaction of Zn²⁺ (0-20 mM) with H_4_DOBDC (0.5 mg mL^-1^) was assessed under identical conditions, and the fluorescence spectra were recorded using the same spectrometer (Ex = 376 nm). The fluorescence quenching parameters obtained from these measurements were used to calculate the Stern-Volmer constant (K_sv_) via the Scatchard and Stern-Volmer equations, thereby allowing for the evaluation of the binding strength between Zn²⁺ and both AChE and H_4_DOBDC.

**Limit of detection (LOD) determination**

The limit of detection (LOD) was defined as the lowest analyte concentration within the linear range that produces a measurable and distinguishable response from the blank under the experimental conditions. In this work, the LOD was determined as the minimum concentration at which a reproducible colorimetric signal could be visually observed and quantitatively differentiated from the background based on the corresponding calibration curve

**Porosity calculation of aerogel**

For the porosity evaluation, we adopted a commonly used method^[1]^. The porosity (P) of the aerogel was estimated based on the total pore volume and the overall volume of the aerogel monolith (V_m_). The total pore volume (V_P_) consists of the intrinsic pore volume of the MOF component (V_P-MOF_) and the interparticle (external) pore volume (V_P-Ext_), expressed in Equation S1:

$$P\left( \% \right)=\frac{V_{P}}{V_{m}}\times100=\frac{V_{P-MOF}+V_{P-Ext}}{V_{m}}\times100$$

The interparticle pore volume (V_P-Ext_) was further estimated by subtracting the solid volumes of both MOF and Ca-alginate (CA) matrix from the total aerogel volume, as expressed in Equation R2:

$$V_{P-Ext}=V_{m}-V_{MOF}-V_{CA}$$

Therefore, the porosity can be rewritten as Equation R3:

$$P(\%)=\frac{V_{P-MOF}+V_{m}-V_{MOF}-V_{CA}}{V_{m}}\times100$$

Here, V_p-MOF_ was obtained from the pore volume derived from BET measurements and normalized based on the corresponding MOF mass. The total volume of the aerogel monolith (V_m_) was approximated from the volume of the hydrogel prior to freeze-drying, assuming negligible volume change. V_MOF_ and V_CA_ represent the solid volumes of MOF particles and Ca-alginate (CA) matrix, respectively, which were calculated based on their mass and intrinsic densities. The calculated porosity values are summarized in Table S3.

**Based on smartphone-based aerogel digital biosensors**

To minimize environmental factors (such as relative positioning between samples and the camera, and intensity of the light source) and operational errors (information extraction and conversion), we conducted tests with the sample positioned at specified locations relative to the recorder. The light source was positioned 25 cm above the top of the sample to reduce interference from ambient light. Subsequently, a smartphone was used to capture true-color images of the sample, which were then processed using ImageJ software. The software separated the acquired true-color image into red (R), green (G), and blue (B) channels, with each channel represented by 256 integer values based on brightness. The product of R intensity and B intensity was digitally processed, and the resulting R×B channel was subjected to image mapping to obtain pseudo-colors.

**Recovery rate calculation**

The recovery rate refers to the percentage of an analyte that is accurately measured or detected by a sensor after being added to a sample. The recovery rate is calculated using the following formula:

$$Recovery rate=\frac{C_{m}}{C_{a}}\times100\%$$

*C_m_* is the actual measured concentration; *C_a_* is the concentration of pesticide added to the sample.


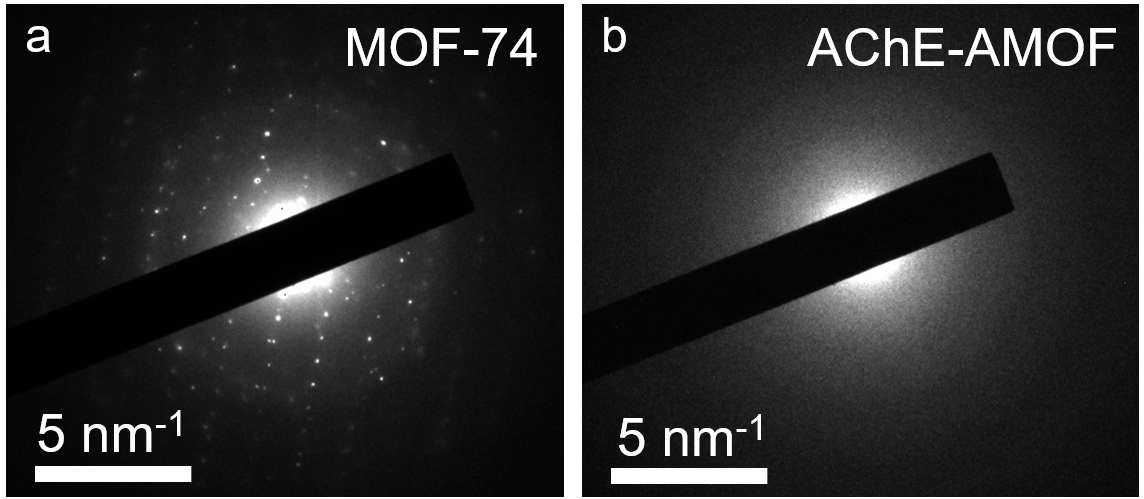


**Fig. S1** Selected area electron diffraction (SAED) patterns of crystalline MOF-74 and AChE-AMOF. MOF-74 exhibits distinct diffraction spots corresponding to its crystalline structure, while AChE-AMOF shows broad diffuse halo-like rings without discrete spots, indicating its amorphous nature.


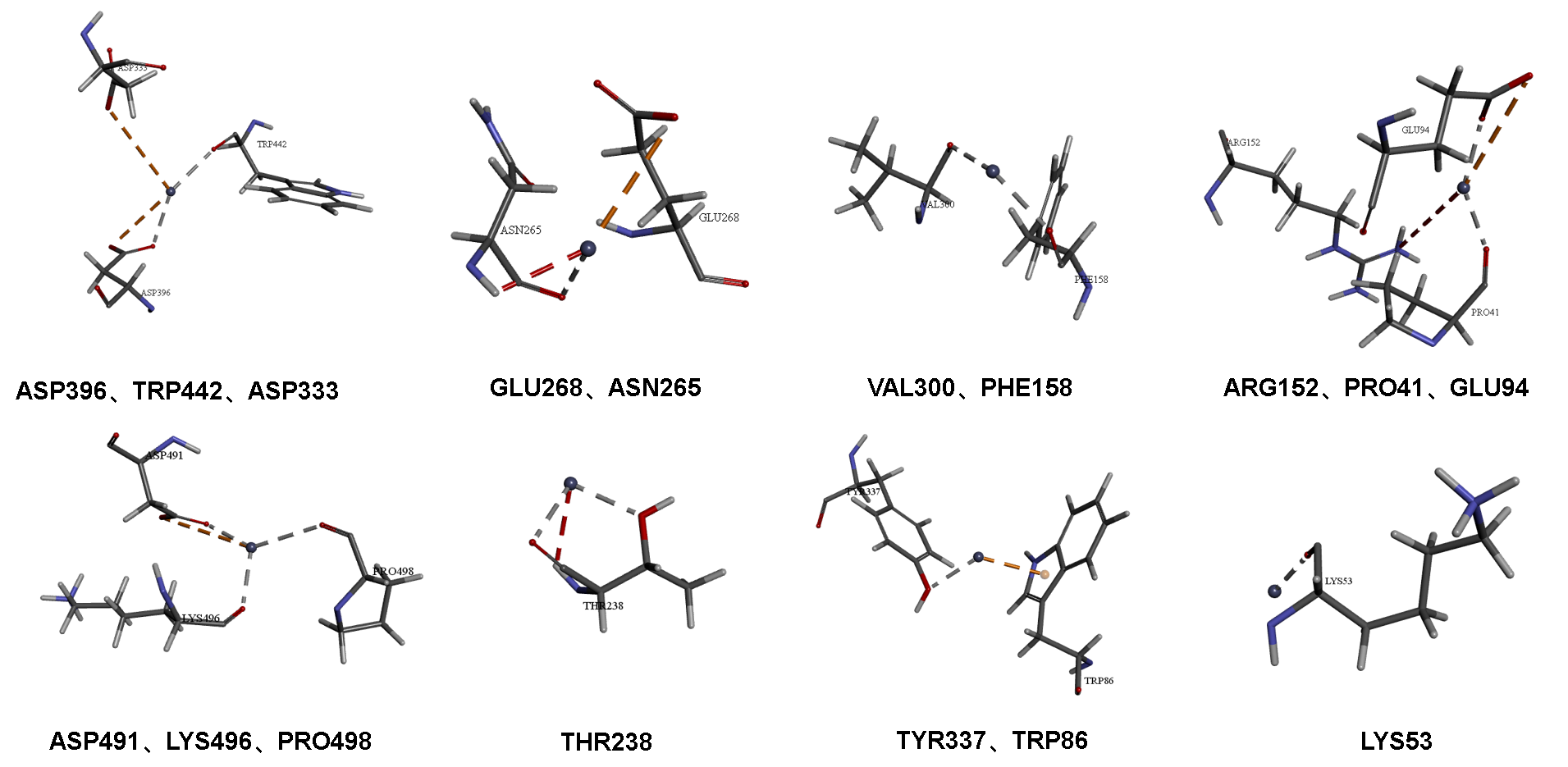


**Fig. S2** The possible key binding sites for AChE and Zn(II). PDB code:1C2B.

To understand how Zn(II) binds to AChE via coordination bonding, eight possible coordination bonding binding sites between Zn(II) and AChE were identified by molecular docking along with their respective -CDCKER-energies (Fig. 2b).

**
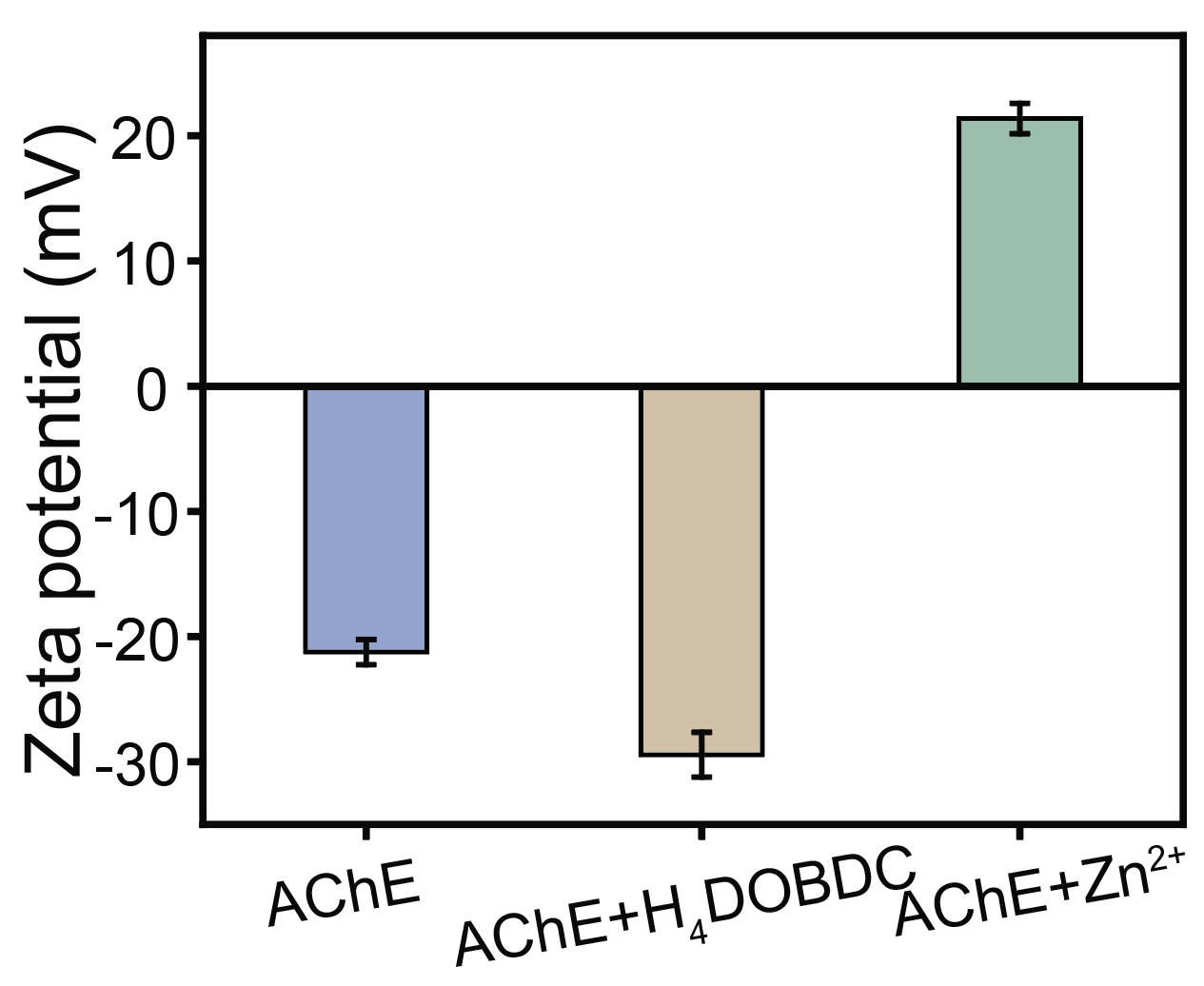
**

**Fig. S3** The Zeta potential of AChE+Zn^2+^, AChE and AChE+H_4_DOBDC.

**
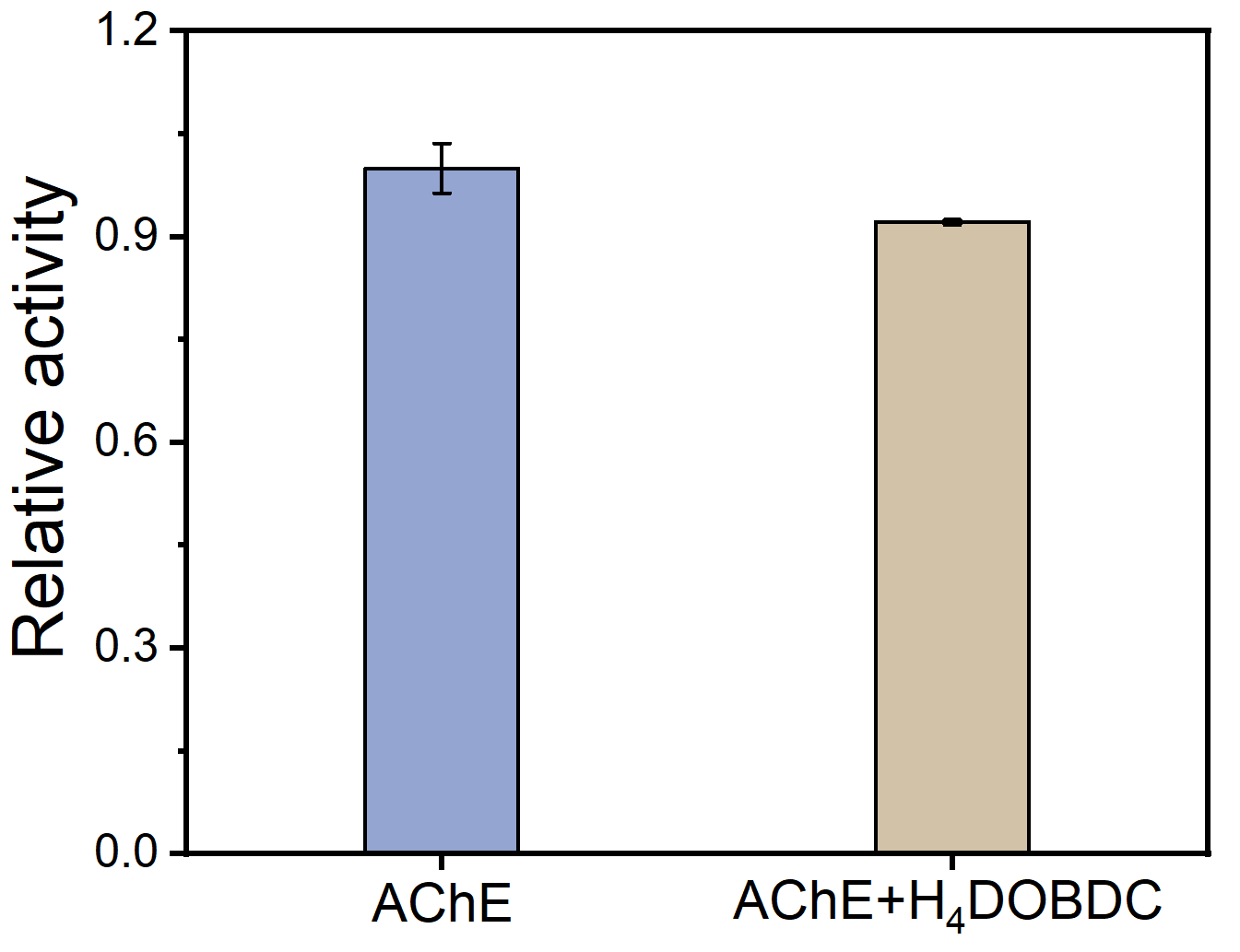
**

**Fig. S4** Relative activity of AChE and AChE+H_4_DOBDC (5 mg mL^-1^) (the free AChE relative activity is defined as 1.0).


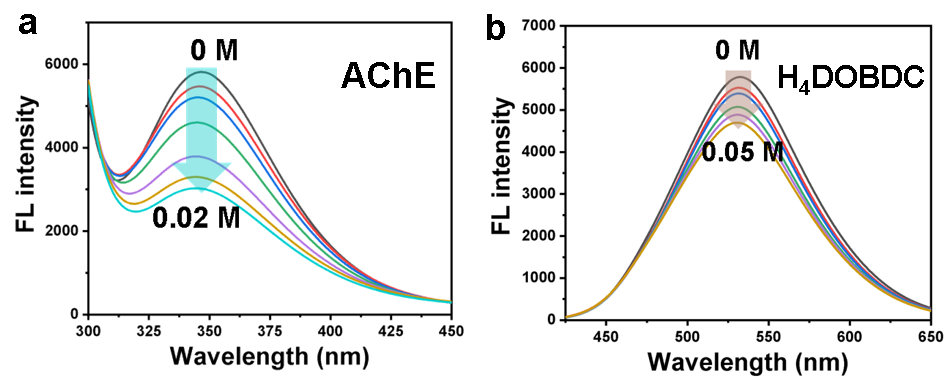


**Fig. S5** Effect of Zn^2+^ concentration on the fluorescence spectra of AChE and H_4_DOBDC.

Fluorescence spectroscopy is widely used to study interactions between fluorescent substances (such as proteins, ligand, etc) and small molecules due to its selectivity, high sensitivity, and non-destructive nature^[2]^. To elucidate the interaction mechanisms between Zn^2+^ and either AChE or H_4_DOBDC, we conducted fluorescence spectroscopy analysis. By examining the changes in fluorescence spectra of H_4_DOBDC and the intrinsic chromophores (Trp, Tyr, Phe) in AChE before and after exposure to Zn^2+^ at specific excitation wavelengths, we aimed to determine the binding affinity and interaction mechanisms between Zn^2+^ and AChE or H_4_DOBDC. Fig. S5 illustrates the effect of varying Zn^2+^ concentrations on the fluorescence intensity of AChE and H_4_DOBDC.


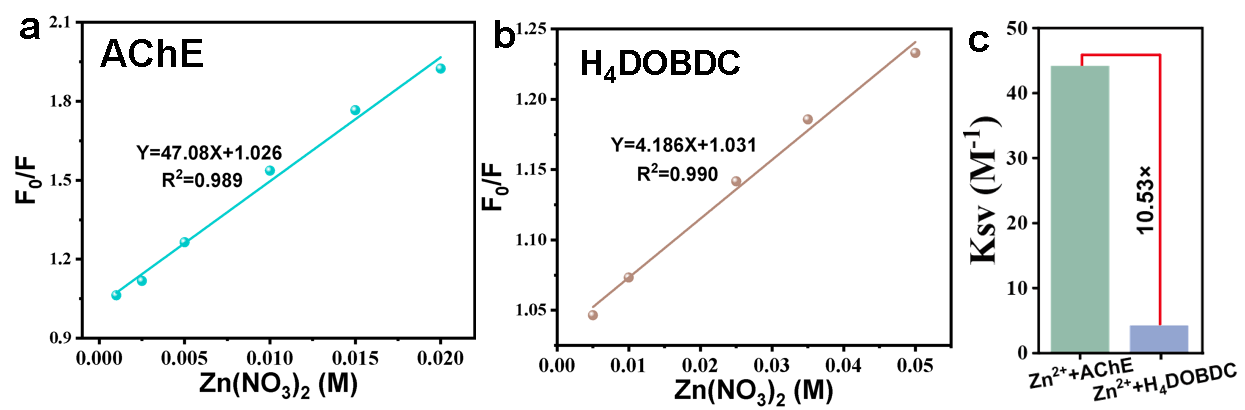


**Fig. S6** Linear fitting results of F₀/F versus Zn(NO_3_)_2_ from the fluorescence spectra of **a)** AChE and **b)** H_4_DOBDC binding with different concentrations of Zn²⁺.

To determine the fluorescence quenching mechanism of Zn^2+^ with AChE and H_4_DOBDC, the Stern-Volmer quenching constant (K_sv_) was calculated based on the fluorescence experimental data (Fig. S7). The Ksv value is calculated using the Stern-Volmer Equation:

$$\frac{F_{0}}{F}=1+Ksv[Q]$$

In the equation, F_0_ represents the fluorescence intensity in the absence of the quencher, F denotes the fluorescence intensity in the presence of the quencher, [Q] is the concentration of the quencher, and Ksv is the Stern-Volmer quenching constant (with units of M^−1^).

The results reveal that the Stern-Volmer quenching constants (K_sv_) for Zn^2+^ with AChE and H_4_DOBDC are 44.08 M^−1^ and 4.19 M^−1^, respectively. The invariance of K_sv_ with respect to varying quencher concentrations indicates that the observed fluorescence quenching is of the static type. This static quenching arises from the interaction and complex formation between Zn^2+^ and the fluorescent molecules. These findings confirm that Zn^2+^ interacts with and forms complexes with both AChE and H_4_DOBDC, with a notably stronger fluorescence quenching between Zn^2+^ and AChE.


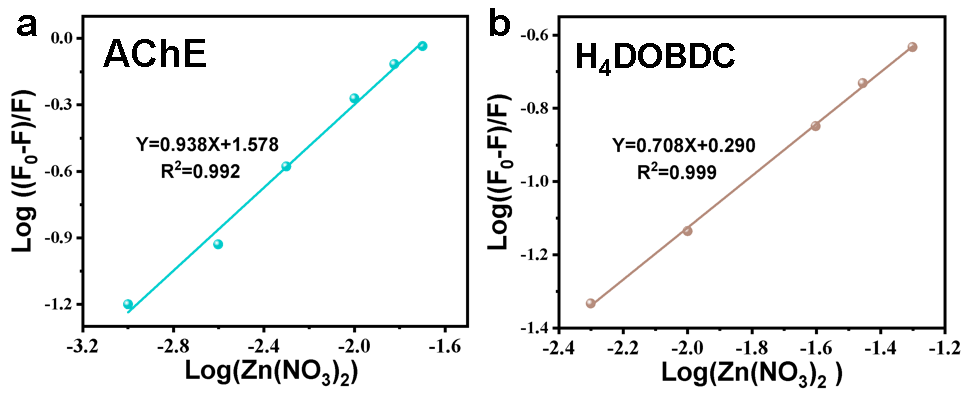


**Fig. S7** Linear fitting results of Log(F_0_-F)/F_0_ versus Log(Zn(NO_3_)_2_) from the fluorescence spectra of **a)** AChE and **b)** H_4_DOBDC binding with different concentrations of Zn^2+^.

To further investigate the binding affinity between Zn^2+^ and AChE or H_4_DOBDC, the association constant (Ka) was calculated based on fluorescence data using the Scatchard equations:

$$Log\frac{F_{0}-F}{F}=LogKa+nLog[Q]$$

In the equation, F_0_ represents the fluorescence intensity in the absence of the quencher, F denotes the fluorescence intensity in the presence of the quencher, [Q] is the concentration of the quencher, n is number of binding sites and Ka is the binding constant, expressed in units of M^−1^.


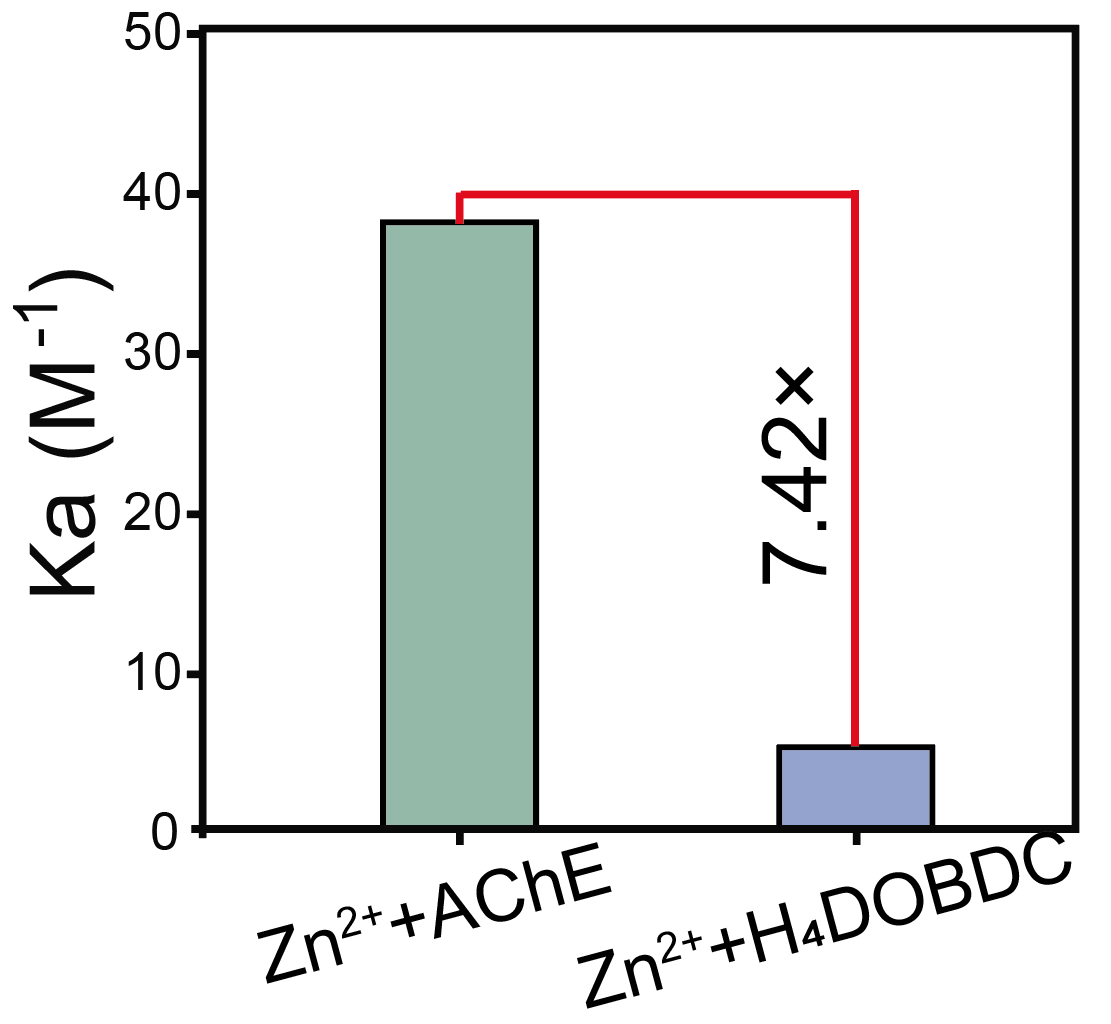


**Fig. S8** K_a_ of Zn^2+^+AChE and Zn^2+^+H_4_DOBDC.


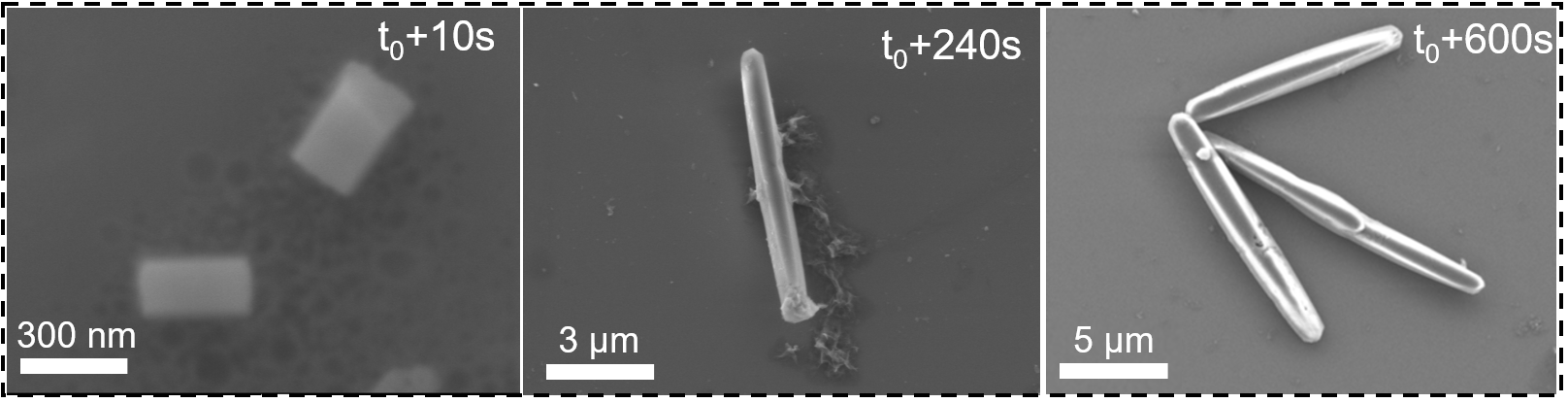


**Fig. S9** SEM images of MOF-74 at different growth times (t_0_ + 10s, 240s, and 600 s).

To further elucidate the formation mechanism of AChE-AMOF composite, the morphological evolution of the assemblies in the presence and absence of AChE was monitored by time-resolved transmission electron microscopy (TEM) and SEM **(Fig. S9** and **S10).** In the absence of AChE, a tetragonal prism-like structure with a size of ≈310 nm already appeared at the early stage within t_0_ + 10 s (t_0_ < 5 s). Upon extending the reaction time to t_0_ + 240 s, these structures grow larger and more elongated via Ostwald ripening, gradually evolving toward a hexagonal morphology. After further growth to t_0_ + 600 s, well-defined hexagonal prismatic structure consistent with crystalline MOF-74 are obtained **(Fig. S10)**. This process follows a typical crystallization pathway involving burst nucleation of tetragonal prism-like intermediates, followed by growth into thermodynamically stable MOF-74 crystals ^[3]^.


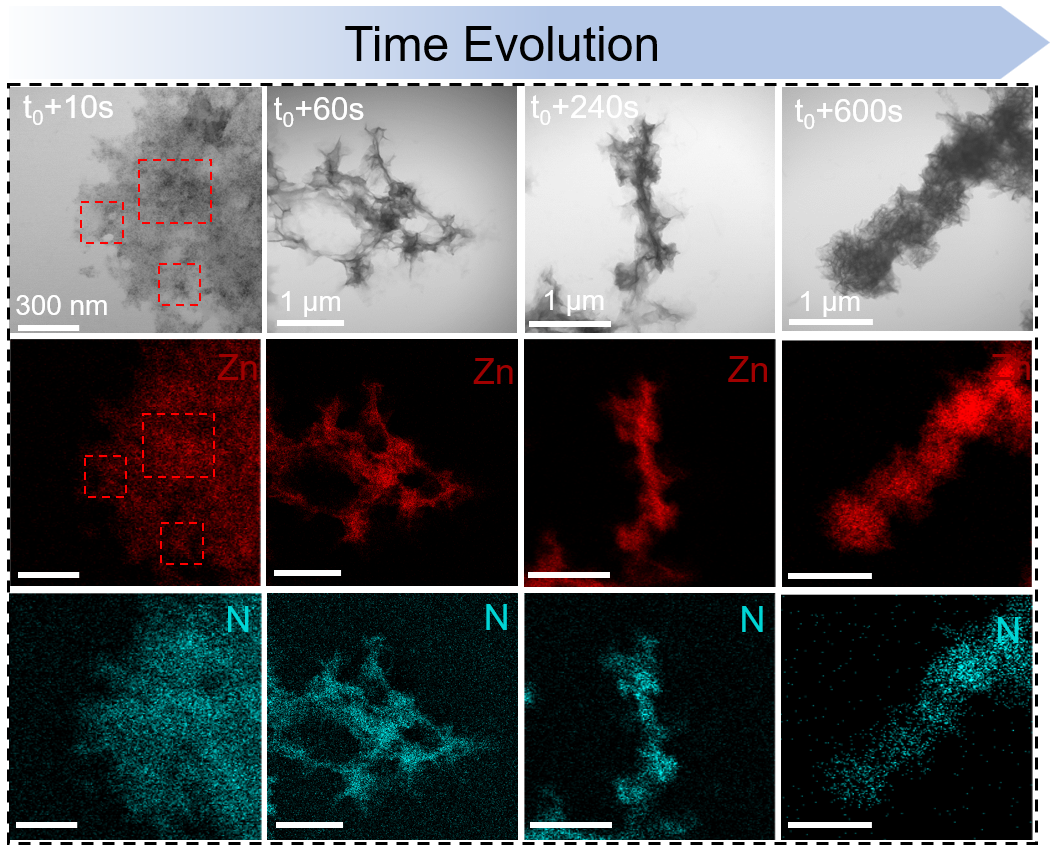


**Fig. S10** TEM images and EDS elemental mapping of AChE-AMOF at different growth times (t₀ + 10 s, 60 s, 240 s, and 600 s), N originates from AChE, while Zn is derived from the MOF-74 framework.

In contrast, in the presence of AChE, amorphous particles rapidly appeared within t_0_ + 10 s. The corresponding elemental mapping results reveal a pronounced local enrichment and inhomogeneous distribution of Zn induced by AChE, which regulates the nucleation of the initial framework **(Fig. S10, red frame)**. At t_0_ + 60 s, these amorphous particles further aggregated to form loosely assembled flower-like nuclei. With prolonged reaction time, the structures evolved into dense polymeric intermediates serving as direct precursors of AChE-AMOF, and finally transformed into amorphous nanoflower-like architectures at t_0_ + 600 s. Based on these observations, we propose that the formation of AChE-AMOF may proceed through two key steps: i) AChE interacts strongly with Zn^2+^, effectively concentrating them on its surface. This localized enrichment disrupts the regular coordination between Zn^2+^ and H_4_DOBDC, thereby promoting the formation of amorphous polymeric precursors; ii) the subsequent growth and aggregation of these polymeric intermediates lead to the formation of the amorphous nanoflower-like AChE-AMOF architecture.

**
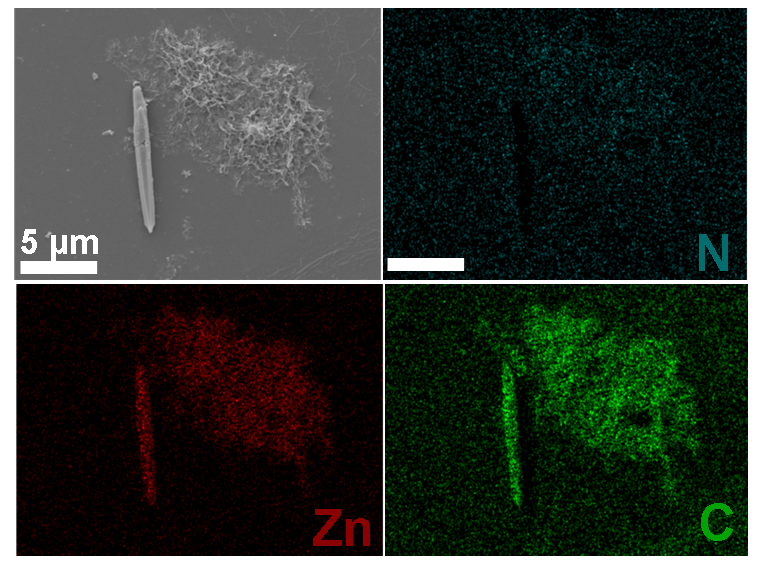
**

**Fig. S11** EDS mapping of MOF-74 with the addition of 0.1 mg of AChE. The N element belongs to the AChE, and Zn is derived from MOF-74.

The EDS images show that the amorphous aggregates contain prominent signals of nitrogen (from AChE), carbon (primarily from H_4_DOBDC), and zinc. This indicates that the amorphous aggregates are likely a ternary complex formed by Zn²⁺, H_4_DOBDC, and AChE.

**
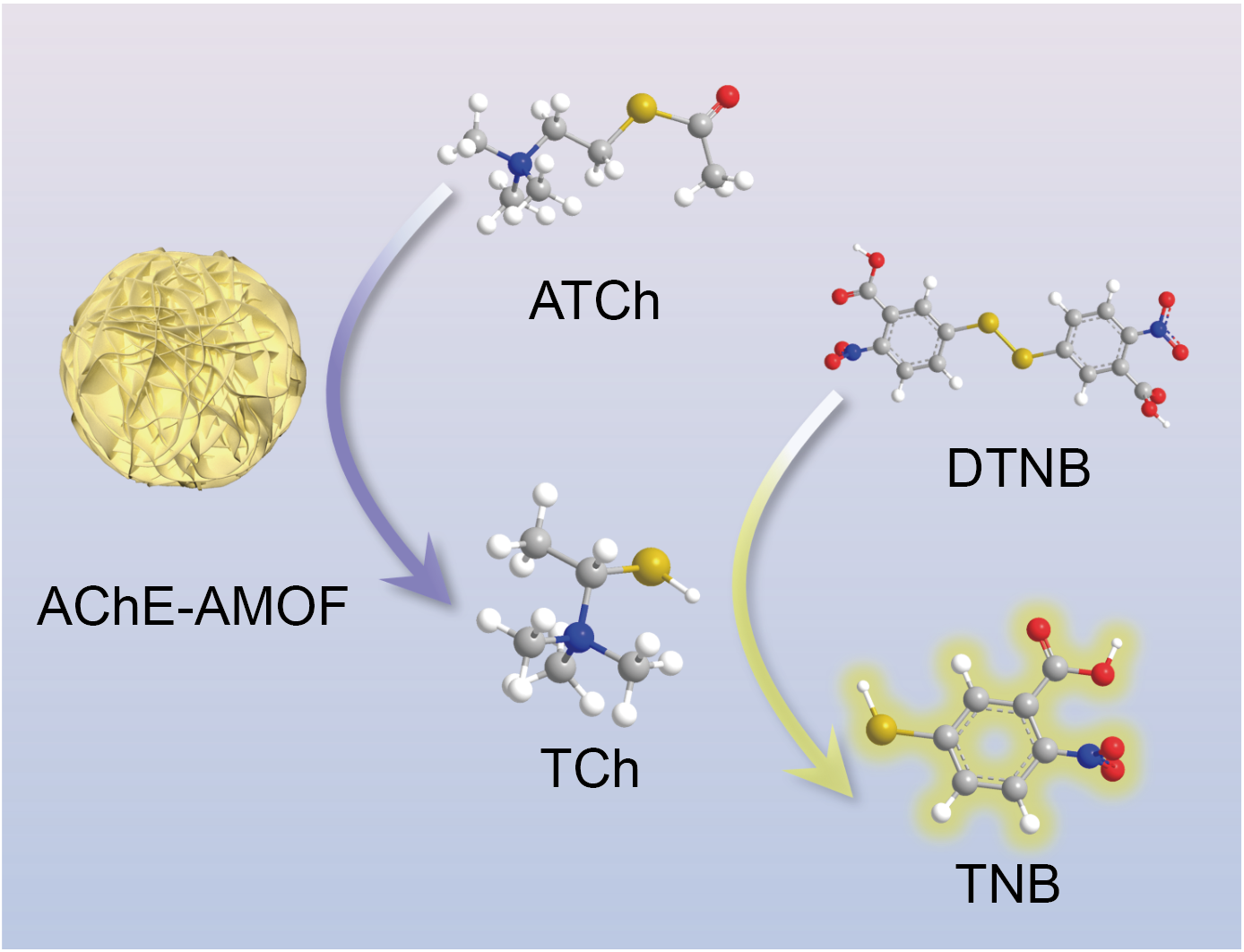
**

**Fig. S12** Schematic illustration of the AChE-AMOF-based sensing mechanism using the DTNB/ATCh colorimetric system.

**
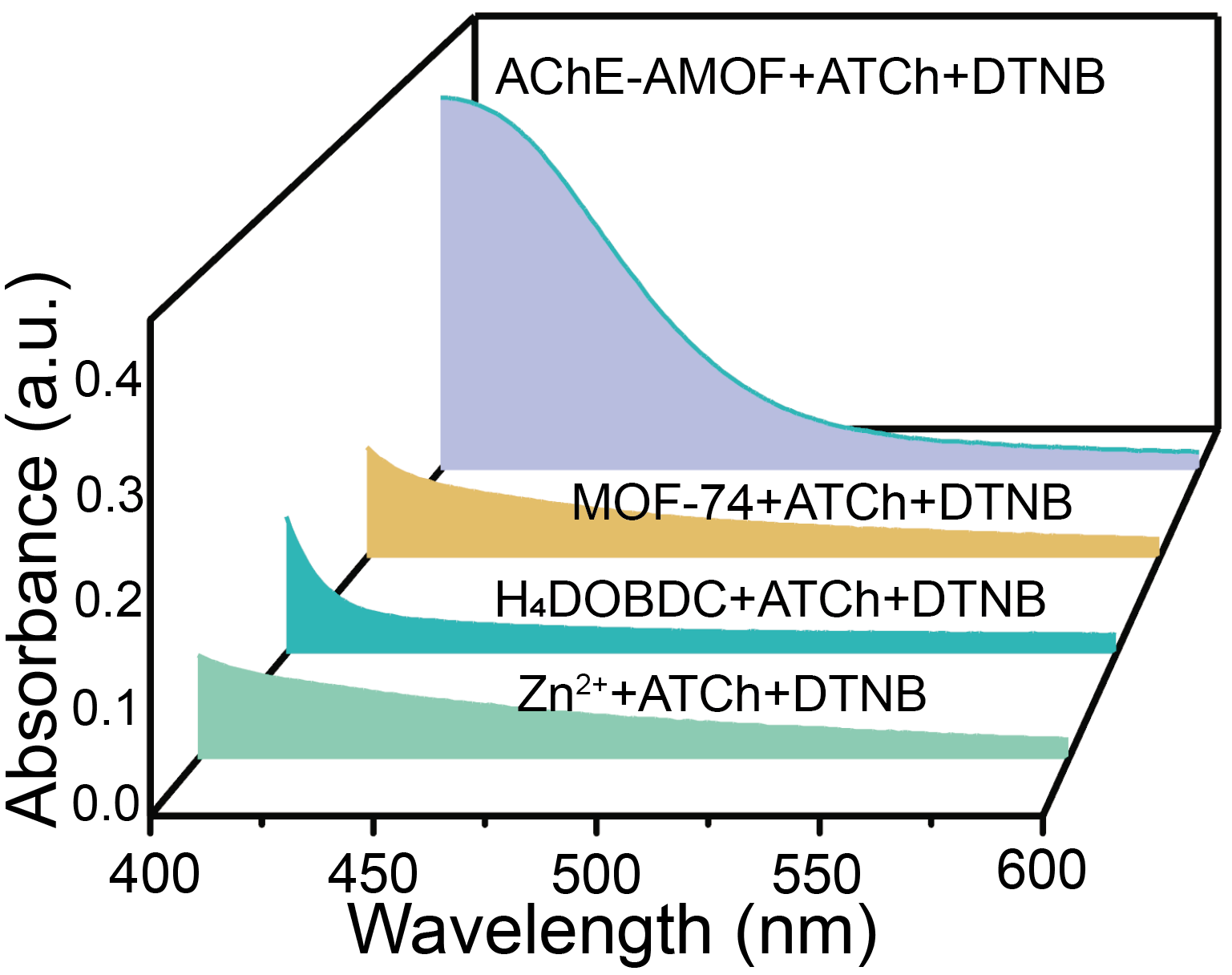
**

**Fig. S13** Feasibility of the AChE-AMOF sensing system, confirming that the absorbance at 412 nm arises from AChE-catalyzed hydrolysis of the chromogenic substrate, with no detectable contribution from the MOF matrix or its precursors.


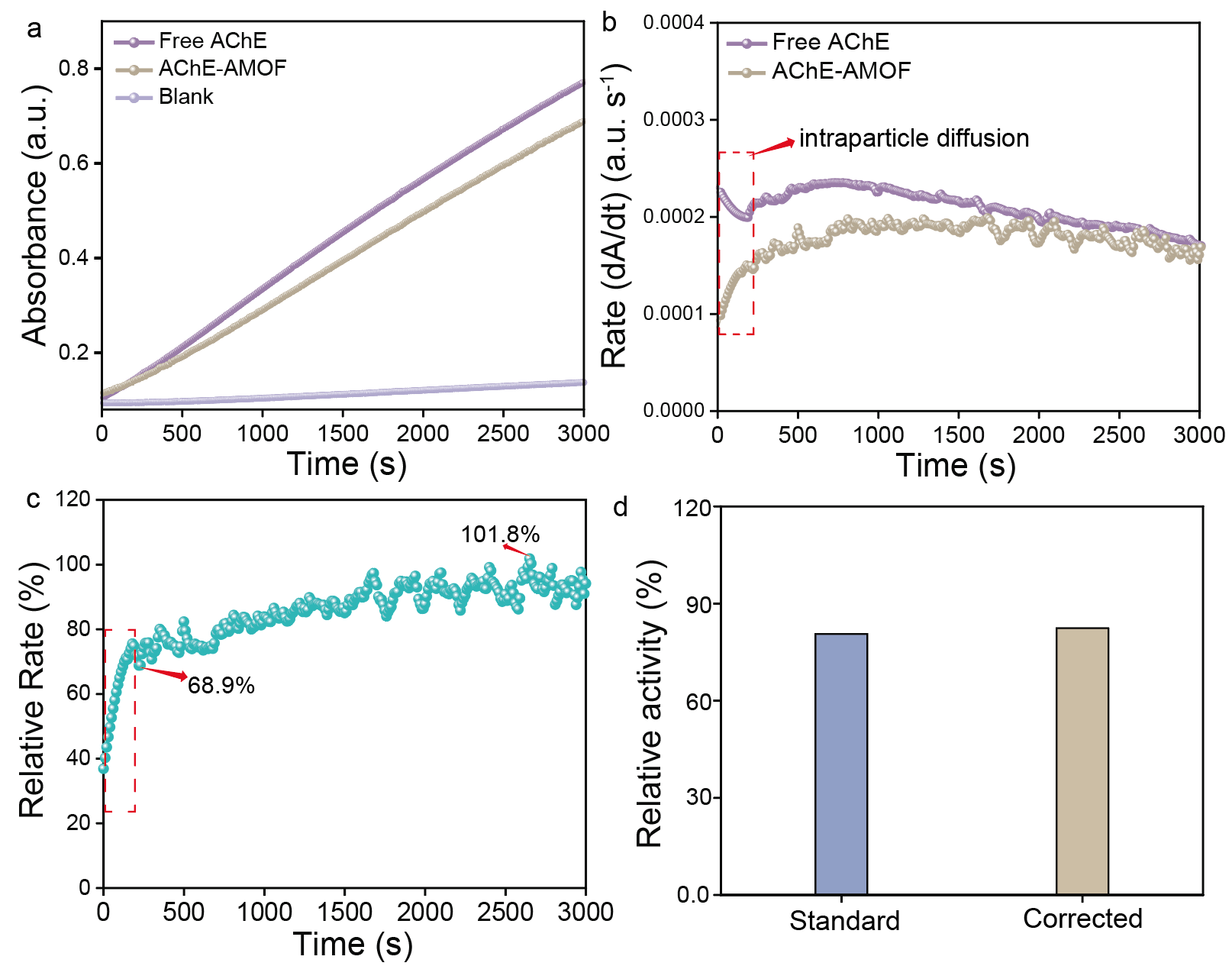


**Fig. S14 a)** Time-dependent enzymatic activity comparison between free AChE and AChE-AMOF. Free AChE (2 mg in 2 mL ultrapure water) and AChE-AMOF (2 mg AChE incorporated into AMOF in 2 mL ultrapure water) were evaluated under identical enzyme feeding conditions. **b)** Instantaneous reaction rates derived from time-resolved absorbance data via numerical differentiation (dA/dt), highlighting the transient and quasi-steady-state regimes. **c)** Time-dependent relative instantaneous reaction rates (%) derived from numerical differentiation of time-resolved absorbance data (dA/dt), defined as the ratio of AChE-AMOF to free AChE. **d)** Comparison between standard activity retention evaluated over 30 min and transient-corrected activity excluding the initial 200 s reaction period.

To evaluate whether the observed activity is influenced by substrate diffusion rather than intrinsic catalytic efficiency, time-resolved absorbance measurements were conducted under substrate-saturated conditions over an extended period (3000 s) **(Fig. S14a)**. After blank subtraction, the instantaneous reaction rates were obtained by numerical differentiation (dA/dt) **(Fig. S14b)**. At the initial stage (t < 200 s), a noticeable difference in the instantaneous reaction rate is observed between free AChE and AChE-AMOF, where free AChE exhibits a higher rate. This indicates that the early-stage reaction is influenced by limited substrate accessibility within the MOF matrix, which is likely associated with substrate diffusion in the porous framework. Therefore, the catalytic behavior during this period does not fully reflect the steady-state enzymatic activity. Importantly, after this initial phase, the reaction rates of both systems gradually stabilize and converge, with the relative rate (%) fluctuating between 68.9% and 101.8%, indicating that a quasi-steady-state regime is reached where mass transfer effects become significantly reduced **(Fig. S14b** and **S14c)**.

In the activity comparison, a standard reaction time of 1800 s (30 min) was used to evaluate catalytic performance, yielding an apparent activity retention of 80.6%. To ensure that the measured activity truly reflects catalytic efficiency, a transient-corrected activity was calculated by excluding the initial 200 s of reaction (i.e., using the 200-1800 s region), which gave a value of 82.4%. The small deviation between the two values (~1.8%) indicates that the initial diffusion stage has a negligible impact on the overall activity evaluation, confirming that the measured activity primarily reflects true catalytic performance **(Fig. S14d)**.

**
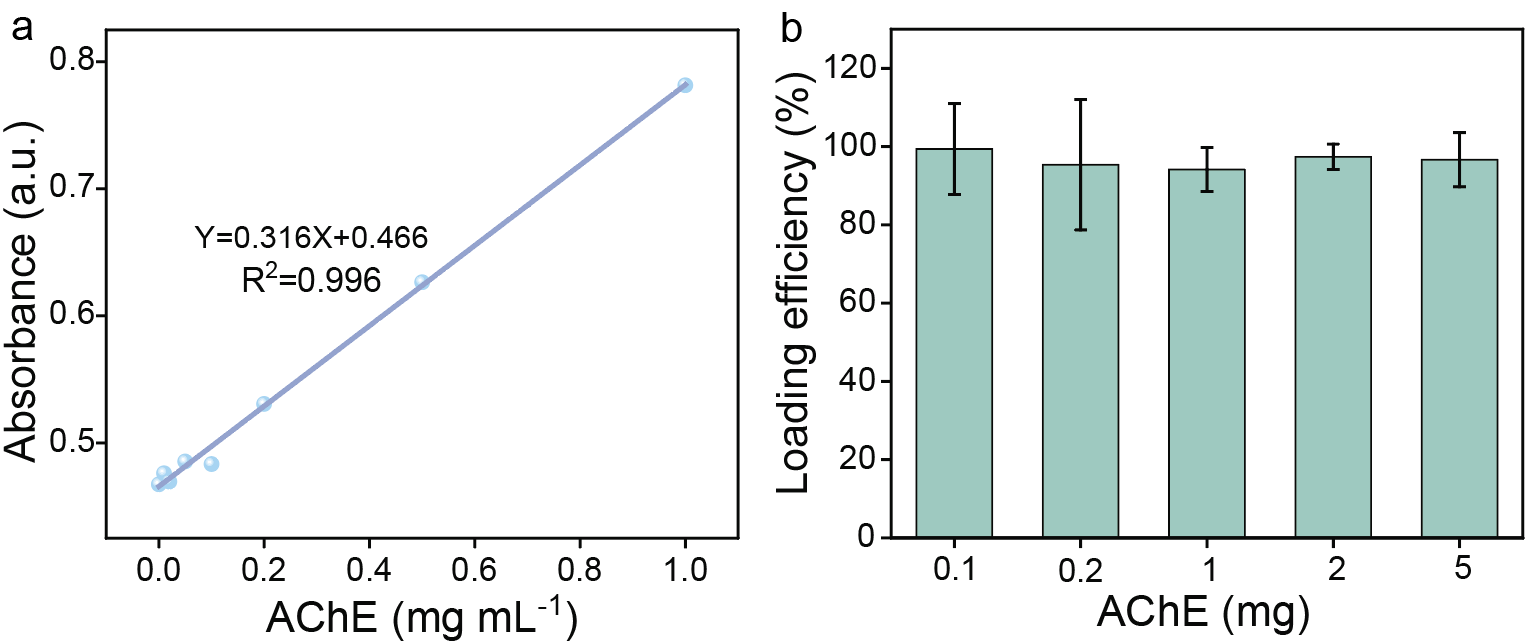
**

**Fig. S15** **a)** Standard calibration curve of AChE concentration based on absorbance at 595 nm measured by the Coomassie Brilliant Blue assay. **b)** Loading efficiency (%) of AChE-MOF prepared with different AChE amounts. AChE-HMOF corresponds to 0.1 mg of AChE, while AChE-AMOF corresponds to 2.0 mg of AChE.


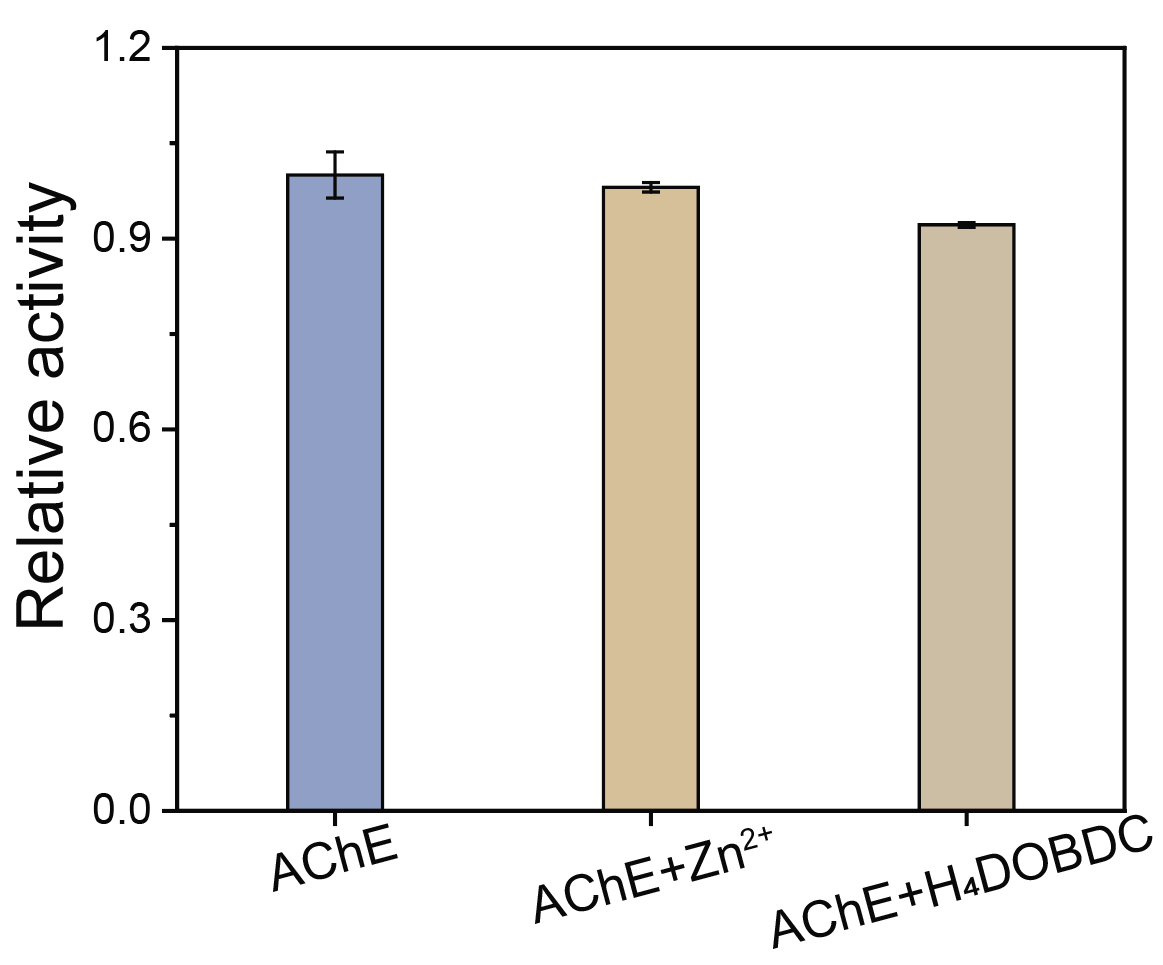


**Fig. S16** Relative activity of free AChE, AChE + Zn^2+^ (25 mM), and AChE + H_4_DOBDC (25 mM). Data are presented as mean ± SD (n = 3 independent measurements).


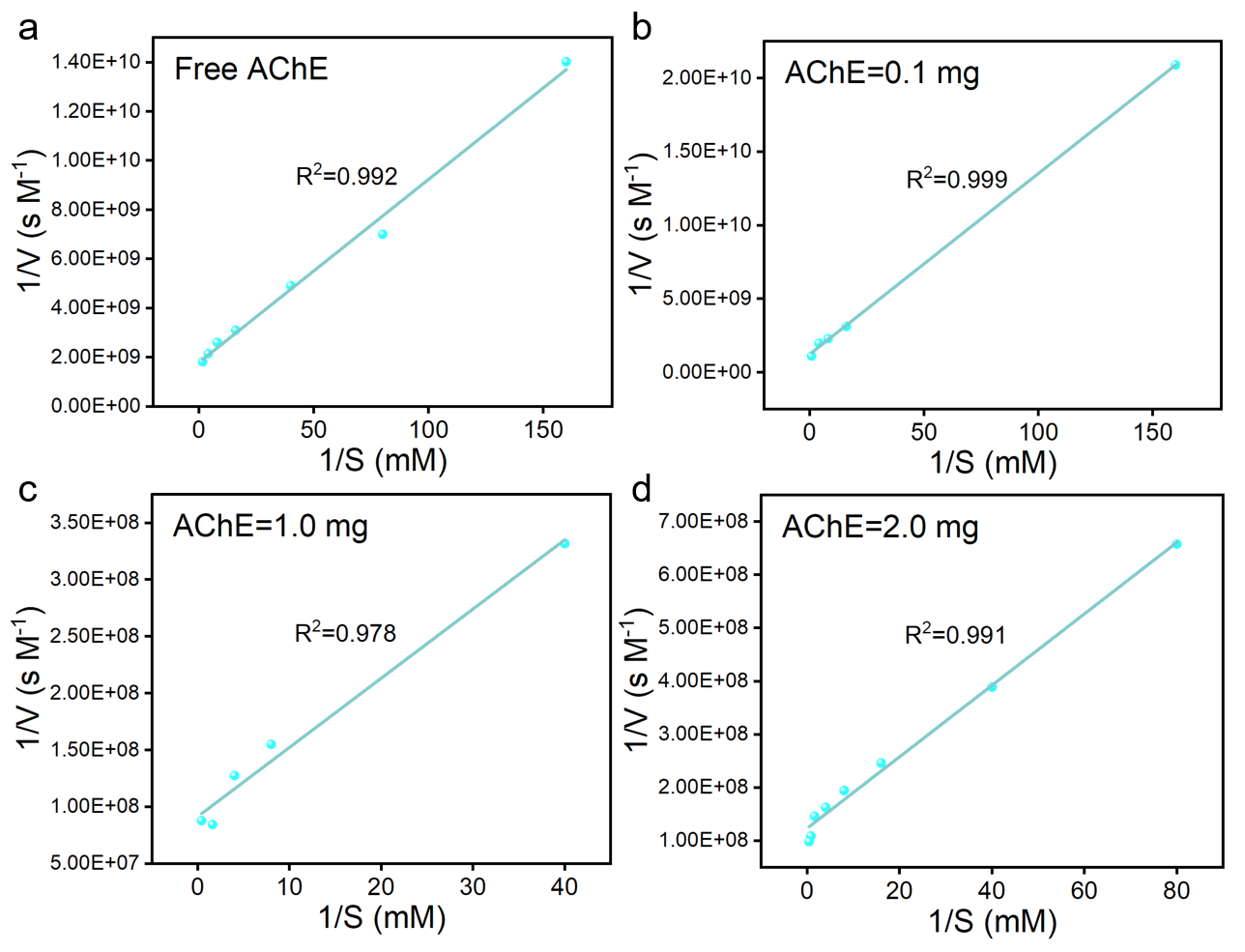


**Fig.S17** Lineweaver-Burk Plots for K_m_ Determination. a) Free AChE, b) AChE-HMOF (AChE=0.1 mg), c) AChE-MOF (AChE=1.0 mg), d) AChE-AMOF (AChE=2.0 mg)


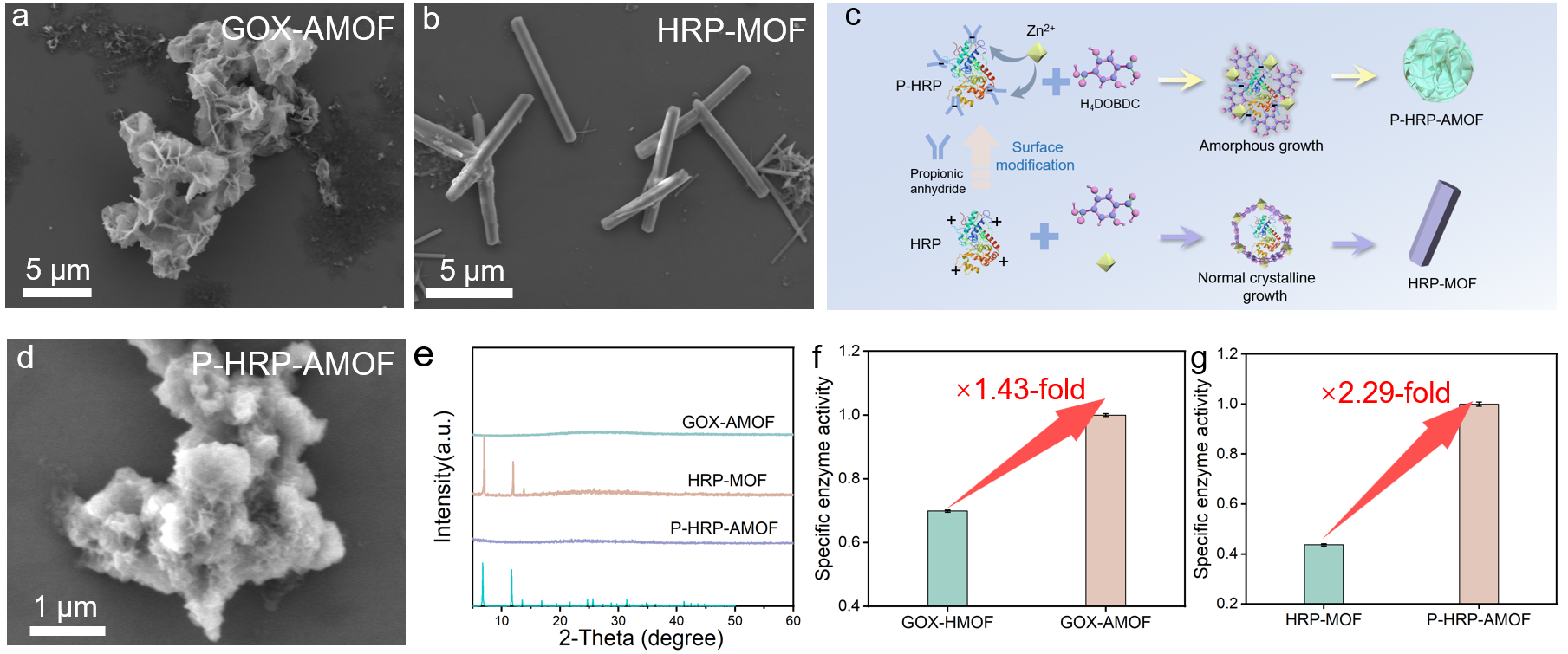


**Fig. S18 a–b)** SEM images of GOX-AMOF and HRP-MOF. The samples were prepared following the same procedure as AChE-AMOF, except that 2 mg of AChE was replaced with 2 mg of GOX or HRP, respectively. **c)** Schematic illustration of MOF growth mediated by surface-modified HRP. Propionic anhydride reacts with the amino groups on the protein surface (primarily the ε-amino groups of lysine residues), forming amide bonds and introducing negatively charged carboxyl groups. This modification converts the overall surface charge of HRP from positive to negative, yielding P-HRP. The negatively charged P-HRP enhances coordination interactions with Zn^2+^, thereby promoting the successful formation of amorphous P-HRP-AMOF. **d)** SEM images of P-HRP-AMOF. The samples were prepared following the same procedure as AChE-AMOF, except that 2 mg of AChE was replaced with 2 mg of P-HRP. **e)** XRD patterns of GOX-AMOF, HRP-MOF and P-HRP-AMOF. **f)** The specific enzymatic activities (defined as the measured activity divided by the total amount of enzyme added in each sample) of GOX-HMOF and GOX-AMOF were compared. GOX-HMOF was synthesized with 0.1 mg GOX, while GOX-AMOF was prepared using 2 mg GOX via an enzyme-mediated strategy. **g)** The specific enzymatic activities of HRP-MOF and P-HRP-AMOF.


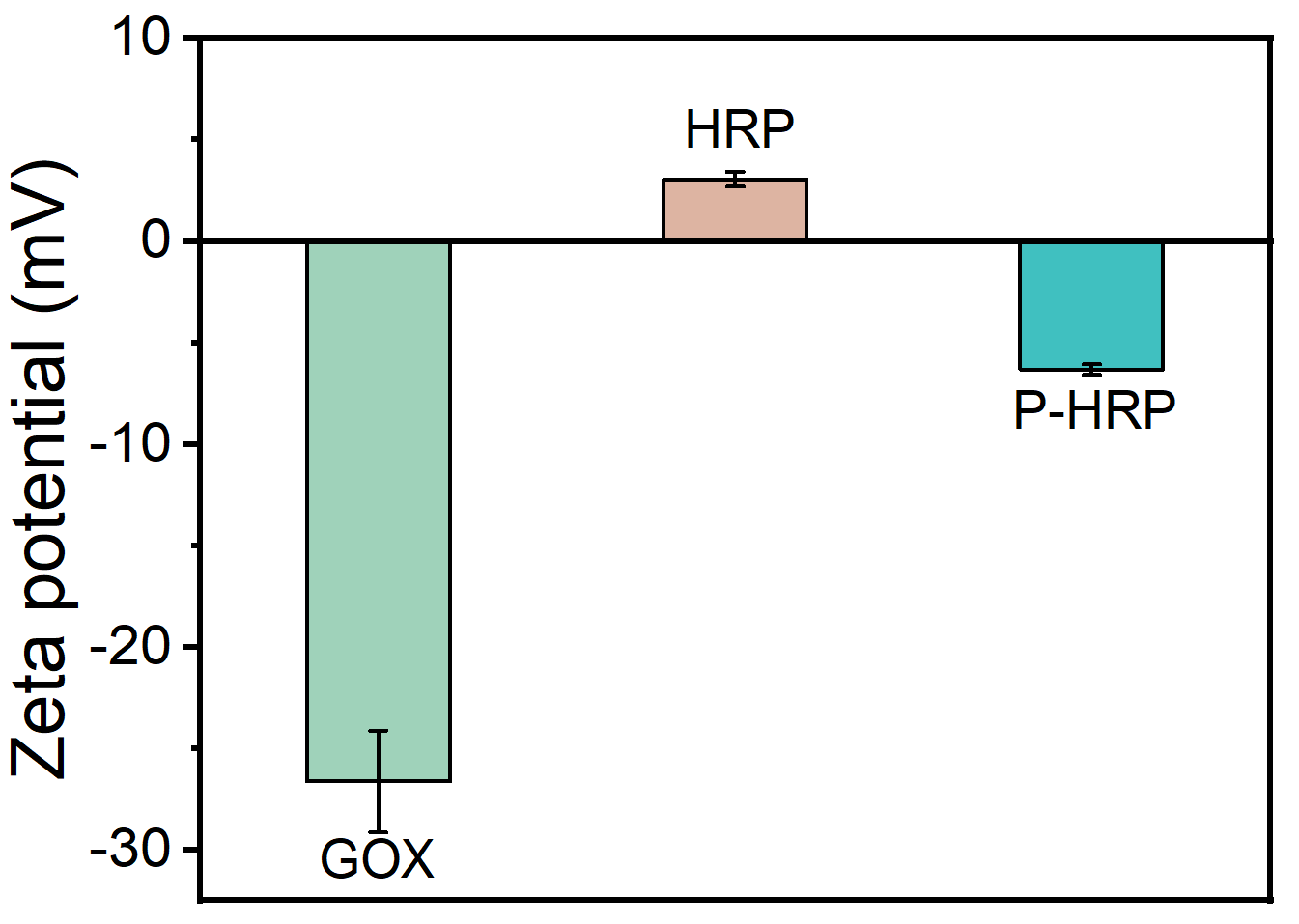


**Fig. S19** The Zeta potential of GOX, HRP and P-HRP.

The zeta potential data indicate that the positively charged HRP was successfully converted to negatively charged P-HRP after modification with acetic anhydride.


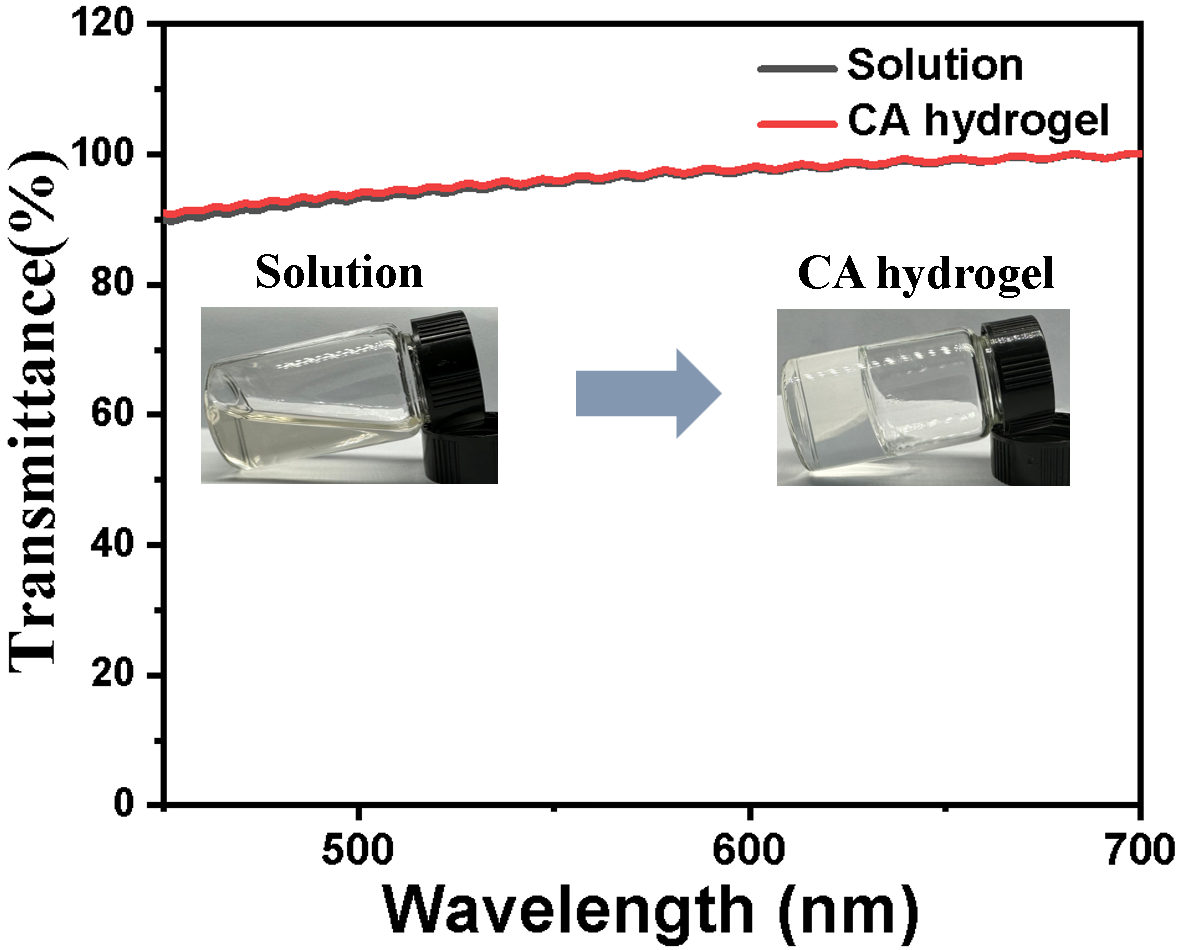


**Fig. S20** Formation of CA hydrogel and transmittance variation.


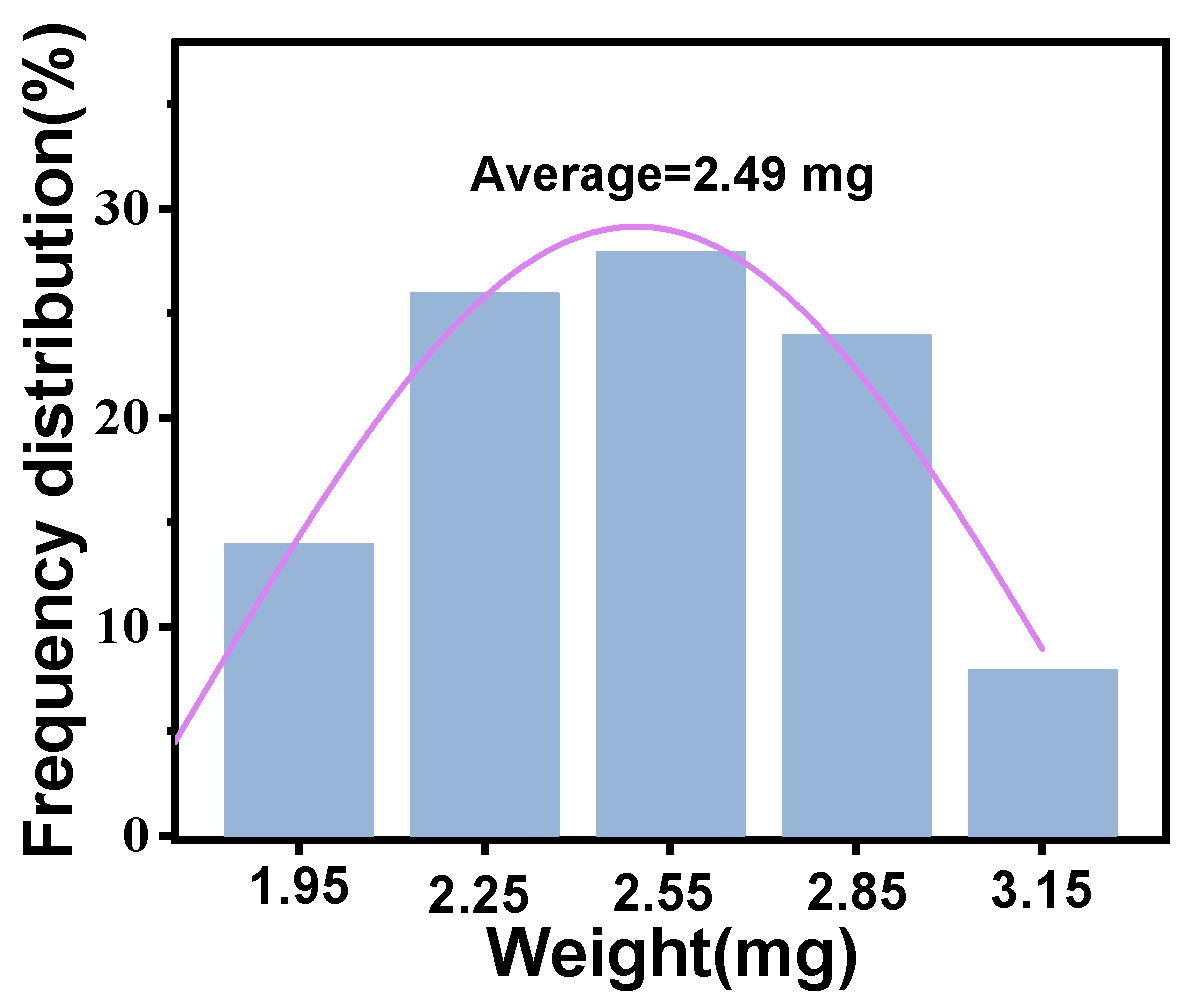


**Fig. S21** The weight distribution of a single aerogel.


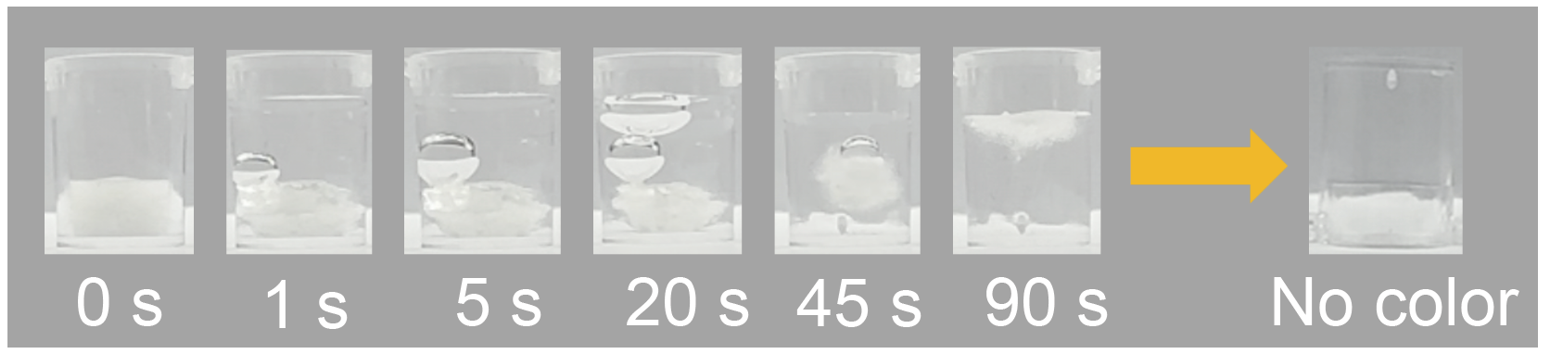


**Fig. S22** Time-dependent photographs of rapid water absorption and swelling behavior of AChE-AMOF-CA hydrogel complex.


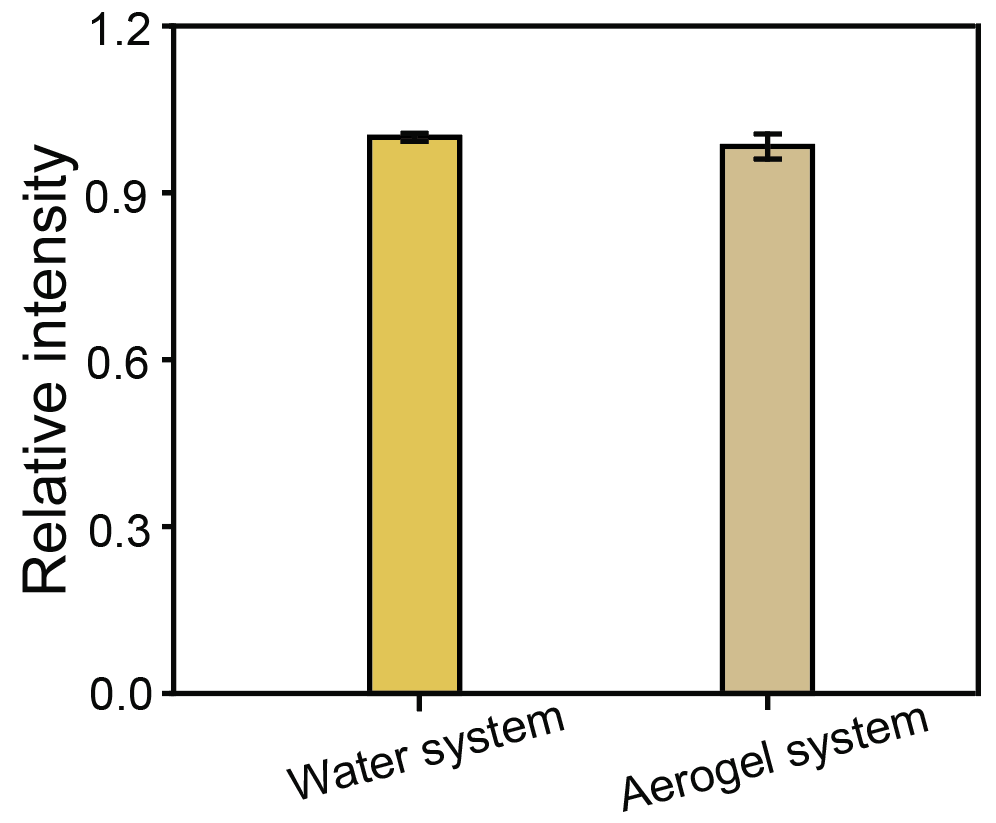


**Fig. S23** Relative intensity of the response of aerogel and water systems to -SH.

**
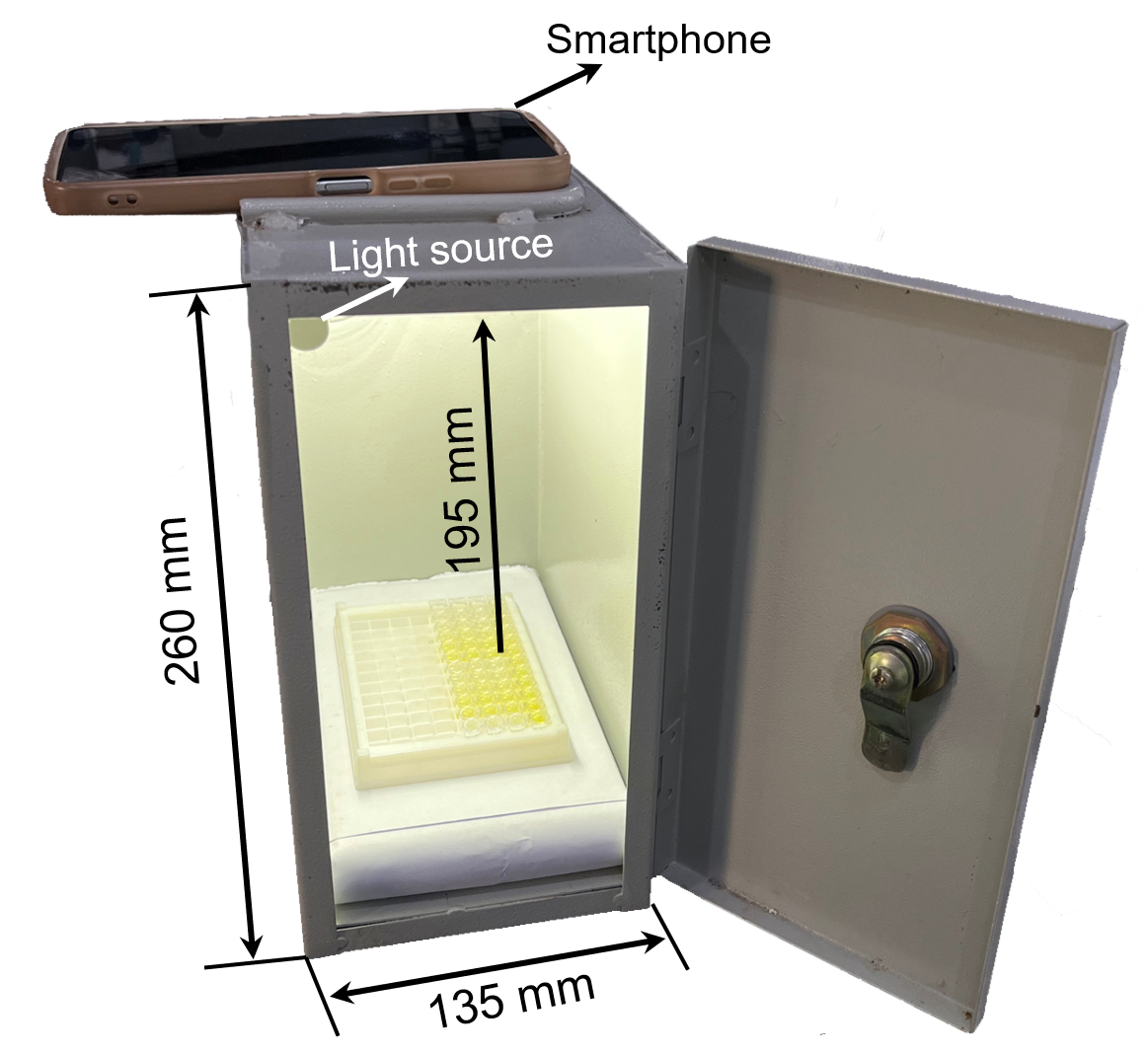
**

**Fig. S24** Photograph of the fixed imaging setup based on a smartphone for standardized colorimetric signal acquisition.

To ensure reliable and reproducible colorimetric signal acquisition, all images were captured under strictly controlled conditions. Specifically, a fixed imaging setup was used, in which the relative positions of the sample, smartphone camera, and light source were kept constant. The distance between the camera and the sample, as well as the illumination intensity, were carefully maintained throughout all measurements to minimize variations arising from environmental light, angle, and exposure differences **(Fig. R24)**. All images were acquired using a smartphone (iQOOZ8v) with fixed camera settings (ISO = 100, exposure time = 1/60 s, and white balance = 5500 K), and the measurements were conducted under a uniform and stable light source to avoid fluctuations in ambient lighting.


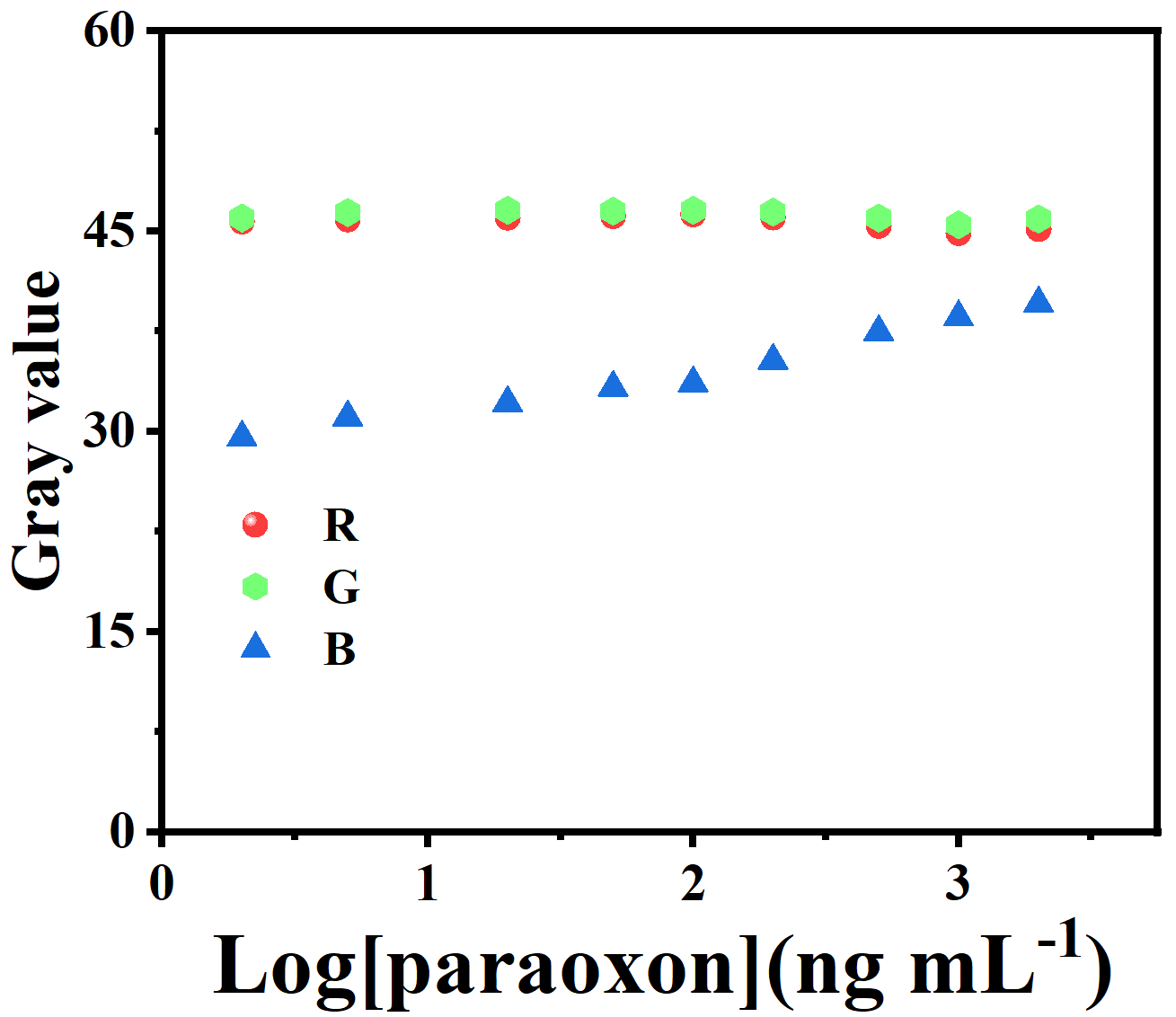


**Fig. S25** The relationship between R, G, B values with Paraoxon concentration


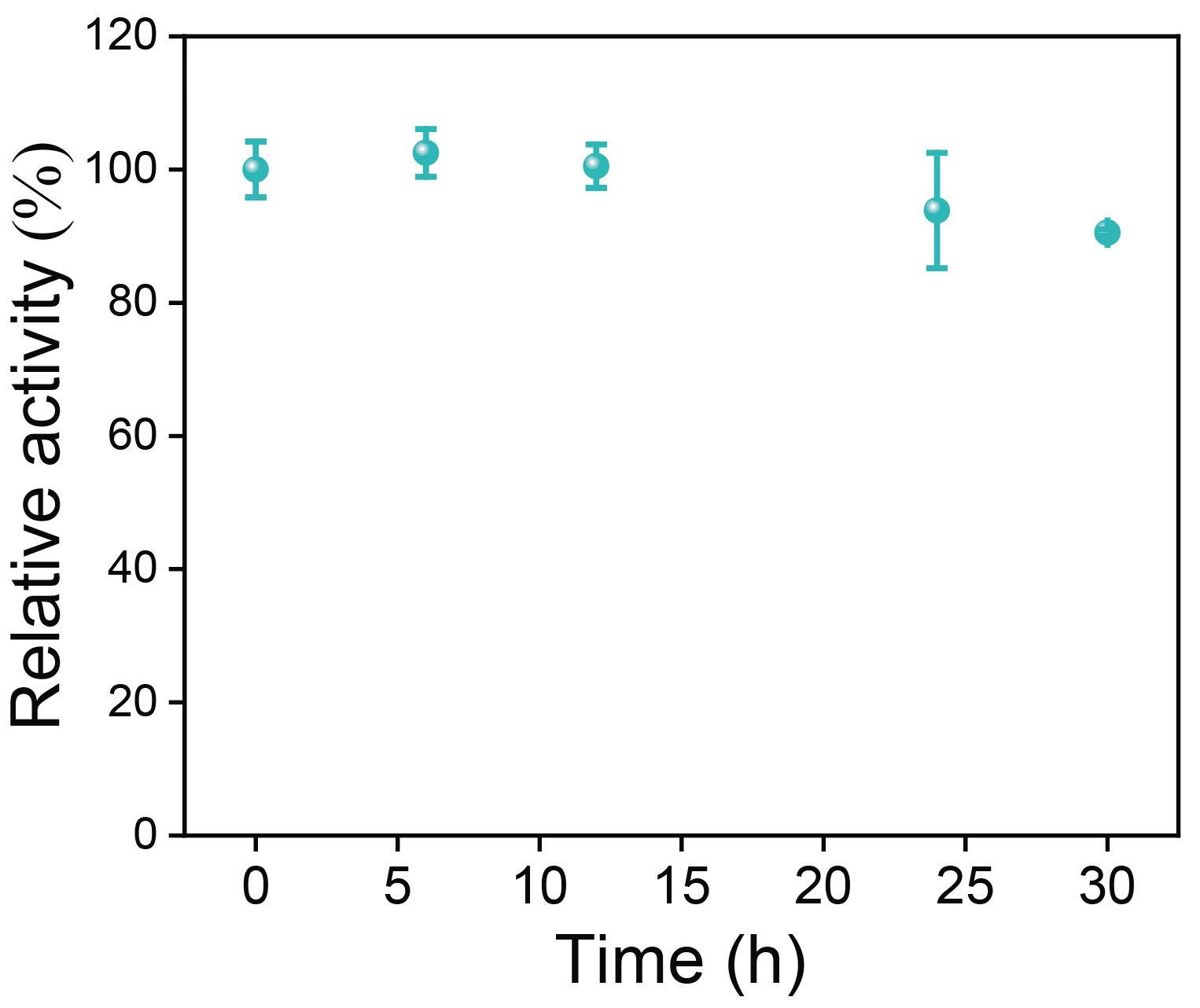


**Fig. S26** Stability of the ADB under ambient light exposure.

**
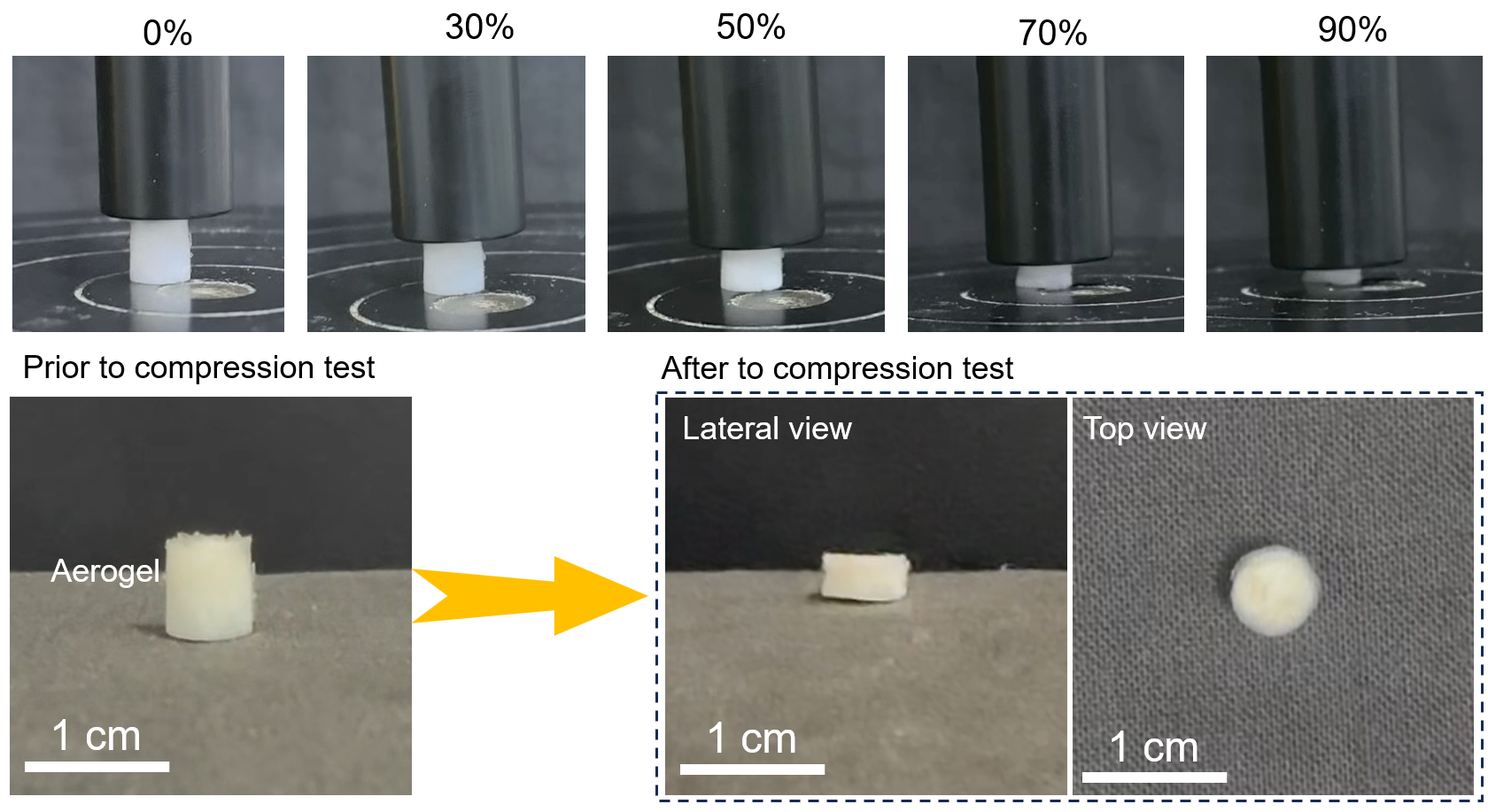
**

**Fig. S27** Images of the uniaxial quasi-static compression test for AChE-AMOF-aerogel.

We evaluated mechanical robustness using uniaxial quasi-static compression tests **(Fig. S27)**. All samples behaved elastically below 30% strain, after which they were plastically compressed to a maximum deformation of 90%, without any structural collapse. At this point, they demonstrated partial recovery, even at maximum deformation. Subsequently, the enzymatic activity of the ADB was measured after applying different compression strains. Compression within the range of 0-90% did not significantly affect the activity of the ADB (variation within 10%) **(Fig. S28)**. These results indicate that the ADB possesses excellent mechanical resilience and maintains stable sensing performance under substantial physical deformation, confirming its suitability for practical handling and operation.

**
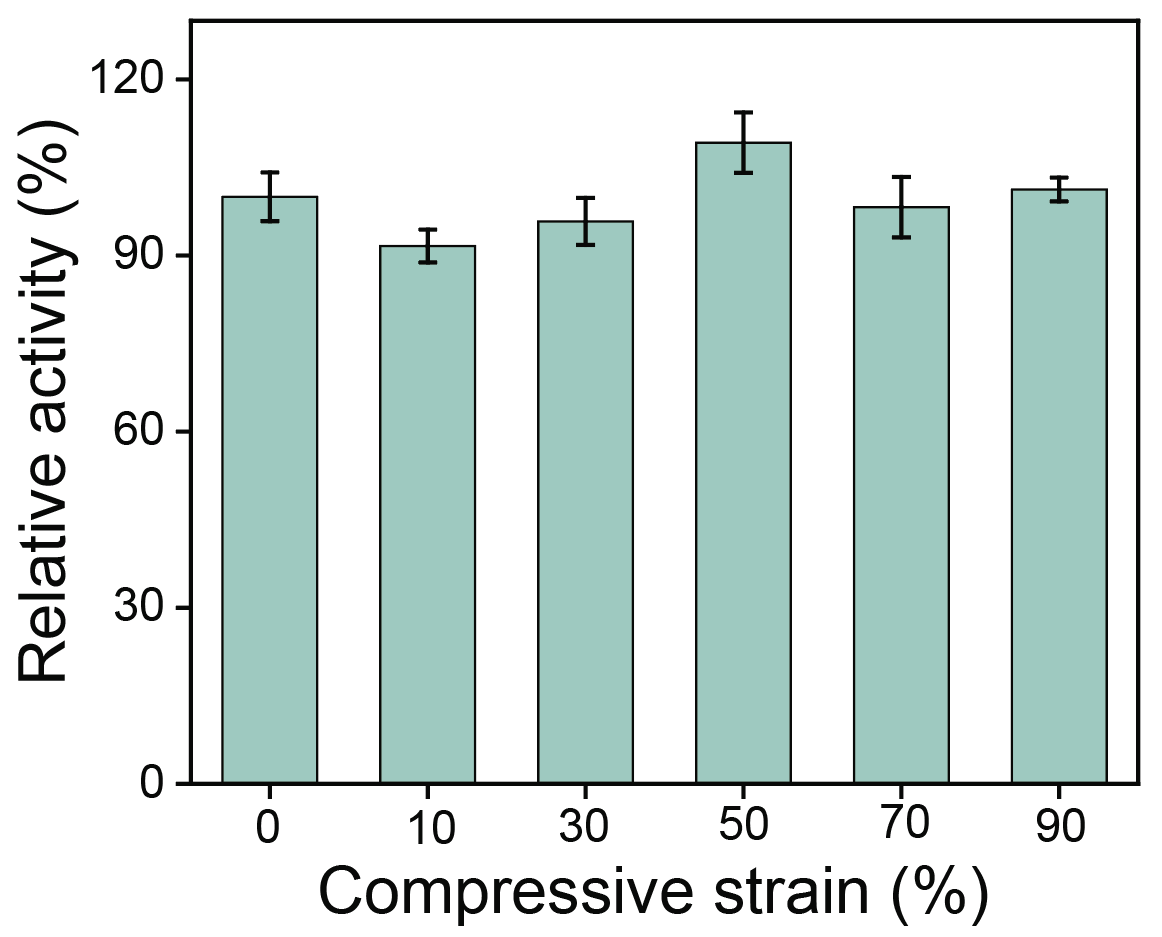
**

**Fig. S28** Comparison of enzymatic activity under different compression strain (%) conditions.


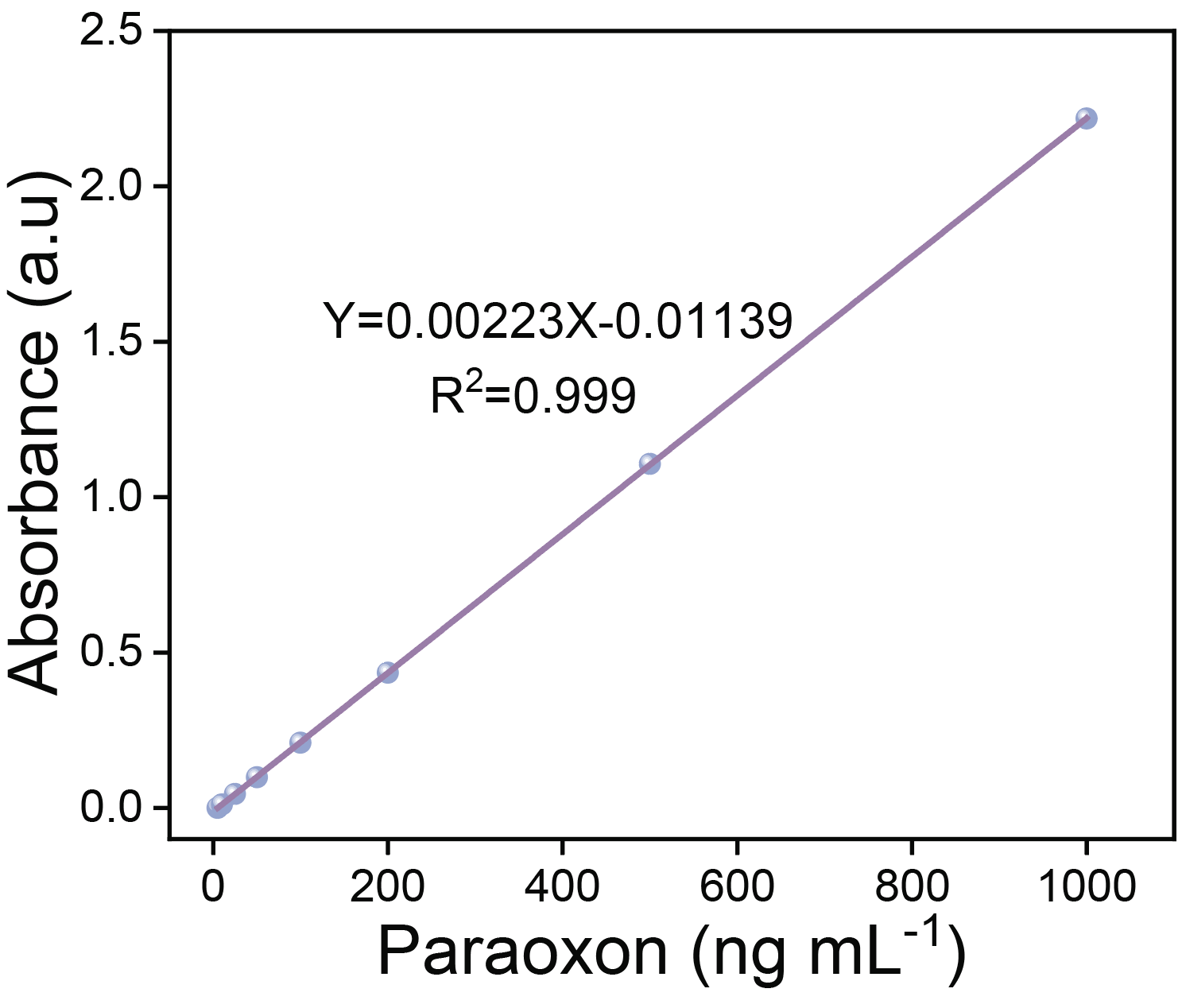


**Fig. S29** Calibration curve constructed from the absorbance at 265 nm versus paraoxon concentration.


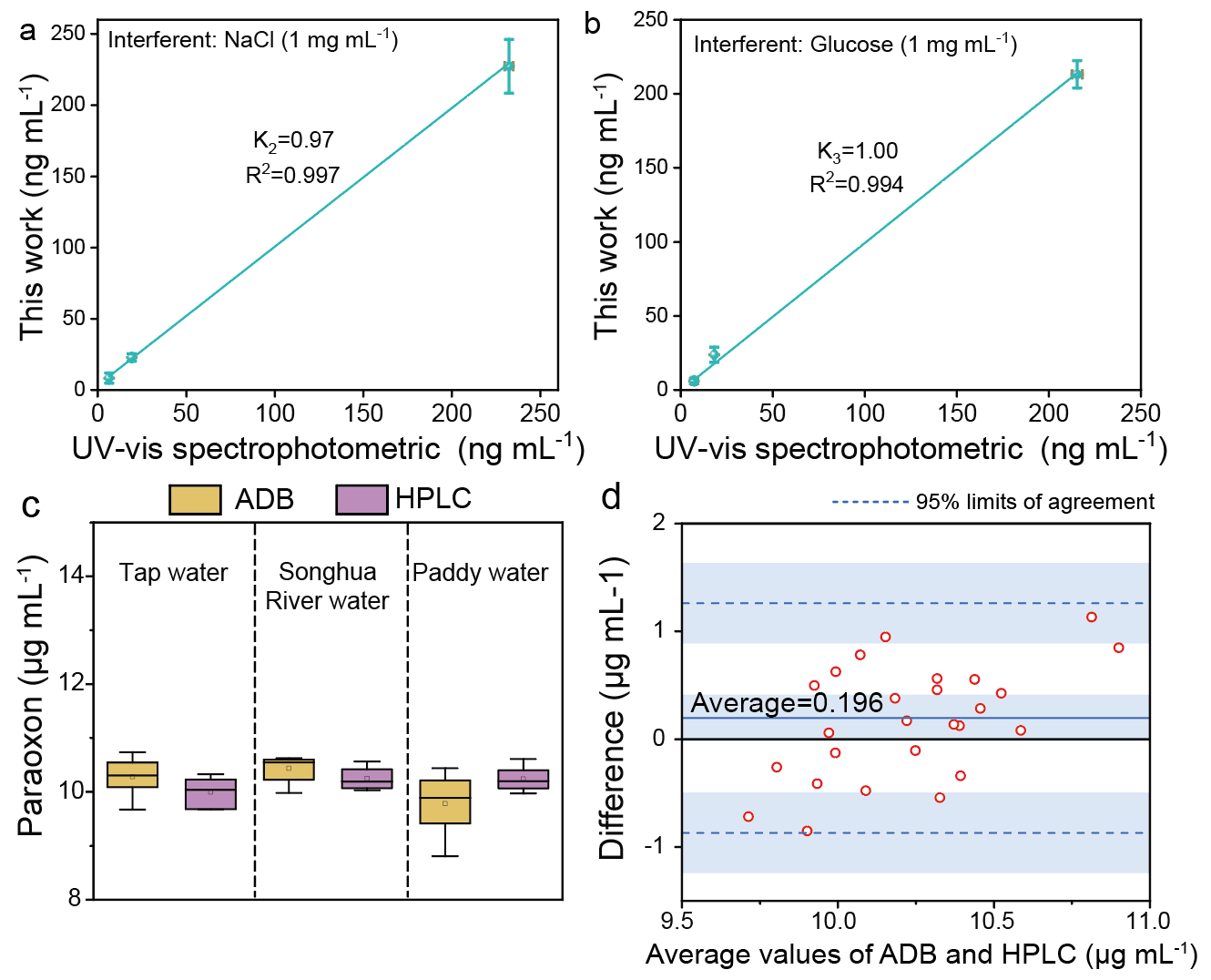


**Fig. S30** Correlation between the ADB-based platform and conventional UV-vis spectrophotometric method for paraoxon quantification under interference conditions. a) In the presence of NaCl (1mg mL^-1^). b) In the presence of glucose (1mg mL^-1^).

**Table S1.** EXAFS fitting parameters.

| Sample | Shell | Bond length  (Å) | CN | σ^2^ (Å^2^) | E0 shift  (eV) | R-factor |
| --- | --- | --- | --- | --- | --- | --- |
| MOF-74 | Zn-O | 2.01±0.03 | 4.0±0.9 | 0.011±0.003 | 2.6±3.6 | 0.020 |
| AChE-AMOF | Zn-N/O | 2.08±0.02 | 4.1±0.7 | 0.007±0.002 | 3.0±1.8 | 0.014 |

[a]. The value of the amplitude reduction factor (S_0_^2^) was fixed to 0.80; [b]. Bond length is the interactomic distance; [c]. CN is the coordination number; [d]. σ^2^ is Debye-Waller factor (a measure of thermal and static disorder in absorber scatter distance); [e]. E_0_  shift is edge-energy shift (the difference between the zero kinetic energy value of the sample and that of the theoretical model); [f]. R factor is used to value the goodness of the fitting.

**Table S2.** The pore size analysis of MOF-74 and AChE-MOF.

| Sample | AChE (mg) | Crystalline state | Pore Size (nm) |
| --- | --- | --- | --- |
| MOF-74 | 0 | Crystalline | 9.819 |
| AChE-HMOF | 0.1 | Crystalline | 11.636 |
| AChE-AMOF | 2 | Amorphous | 21.801 |

**Table S3** Results of the assessment of the porosity of the aerogels.

| Sample | V_MOF_ (μL) | V_CA_ (μL) | V_P-MOF_ (μL) | V_m_ (μL) | P(%) |
| --- | --- | --- | --- | --- | --- |
| AChE-AMOF- aerogel | 0.078 | 2.49 | 0.0022 | 200 | 98.7 |

Here, V_p-MOF_ was obtained from the pore volume derived from BET measurements and normalized based on the corresponding MOF mass. The total volume of the aerogel monolith (V_m_) was approximated from the volume of the hydrogel prior to freeze-drying, assuming negligible volume change. V_MOF_ and V_CA_ represent the solid volumes of MOF particles and Ca-alginate (CA) matrix, respectively, which were calculated based on their mass and intrinsic densities.

**Table S4** Comparison of the performance of different detection methods for OPs.

| Method | Materials | Linear range  (ng mL^-1^) | LOD  (ng mL^-1^) | Ref |
| --- | --- | --- | --- | --- |
| Electrochemistry | Paper-based device | 2-20 | 2 | ^[4]^ |
| Electrochemistry | NiCo_2_O_4_-PAMAM | 49.43-247.14 | 19.77 | ^[5]^ |
| Electrochemistry | CeO_2_ | 24.7-24700 | 14.8 | ^[6]^ |
| Electrochemistry | MXene | 100-10^6^ | 100 | ^[7]^ |
| Fluorescence | TPE-probe | 49.43-247.14 | 19.77 | ^[8]^ |
| Fluorescence | CDs | / | 54.37 | ^[9]^ |
| Fluorescence | C-dots | 10–1000 | 3 | ^[10]^ |
| Fluorescence | AuNCs@MnO_2_ | 5-500 | 5 | ^[11]^ |
| Colorimetric | N-Cu-MOF | 1000-10^9^ | 25.8 | ^[12]^ |
| Colorimetric | Cu-C_3_N_4_ | 24.7-8151 | 3.12 | ^[13]^ |
| Colorimetric | Au NBPs@Fe-MOF | 8-400 | 4 | ^[14]^ |
| Colorimetric | AChE@MOF-74 | 2-2000 | 2 | This work |

**Table S5.** Determination of paraoxon in spiked samples.

| Sample | Spiked levels  (ng mL^-1^) | Recovery (%) | Relative standard  deviations  (n = 3, %) |
| --- | --- | --- | --- |
| Paddy water | 0 | - | - |
|  | 50 | 95.23 | 0.34 |
|  | 200 | 100.42 | 0.79 |
|  | 1000 | 95.97 | 3.46 |
| Songhua River water | 0 | - | - |
|  | 50 | 98.44 | 0.67 |
|  | 200 | 102.07 | 0.48 |
|  | 1000 | 95.85 | 1.17 |
| Coconut water | 0 | - | - |
|  | 50 | 96.88 | 0.86 |
|  | 200 | 104.72 | 0.71 |
|  | 1000 | 95.89 | 2.71 |
| Milk | 0 | - | - |
|  | 50 | 98.77 | 1.02 |
|  | 200 | 105.16 | 0.98 |
|  | 1000 | 98.78 | 1.31 |

**References**

[1] J. Á. Martín‐Illán, D. Rodríguez‐San‐Miguel, O. Castillo, G. Beobide, J. Perez‐Carvajal, I. Imaz, D. Maspoch and F. Zamora, Angew. Chem. Int. Ed. **2021**, 60(25), 13969–13977.

[2] K. P. L. Kuijpers, C. Bottecchia, D. Cambié, K. Drummen, N. J. König and T. Noël, Angew. Chem. Int. Ed. **2018,** 57, 11278.

[3] Y. Fu, Y. Yao, A. C. Forse, J. Li, K. Mochizuki, J. R. Long, J. A. Reimer, G. De Paëpe and X. Kong, Nat. Commun. **2023**, 14, 2386.

[4] V. Caratelli, G. Fegatelli, D. Moscone and F. Arduini, Biosens. Bioelectron. **2022**, 205, 114119.

[5] Y. Yang, S. Hao, X. Lei, J. Chen, G. Fang, J. Liu, S. Wang and X. He, J. Hazard. Mater. **2022**, 428, 128262.

[6] Y. Sun, J. Wei, J. Zou, Z. Cheng, Z. Huang, L. Gu, Z. Zhong, S. Li, Y. Wang and P. Li, J. Pharm. Anal. **2020**, 220, 114841.

[7] F. Zhao, Y. Yao, C. Jiang, Y. Shao, D. Barceló, Y. Ying and J. Ping, J. Hazard. Mater. **2019**, 384, 121358.

[8] J. Wang, J. Zhang, J. Wang, G. Fang, J. Liu and S. Wang, J. Hazard. Mater. **2020**, 389, 122074. [9] M. M. F. Chang, I. R. Ginjom and S. M. Ng, Sens. Actuators B Chem. **2016**, 242, 1050.

[10] B. Lin, Y. Yan, M. Guo, Y. Cao, Y. Yu, T. Zhang, Y. Huang and D. Wu, Food Chem. **2017**, 245, 1176 .

[11] H. Li, R. Zou, C. Su, N. Zhang, Q. Wang, Y. Zhang, T. Zhang, C. Sun and X. Yan, J. Hazard. Mater. **2022**, 432, 128660.

[12] Z. Yisong, Z. Xue, L. Xiong, L. Zhiping and G. Faming, Sens. Actuators B Chem. **2022**, 362, 131749.

[13] G. Chang, S. Li, Y. Wang, Q. Ran, Q. Tan, S. Gou, H. Du and S. Xu, Sens. Actuators B Chem. **2023**, 398, 134584.

[14] Y. Wang, M. Li, Z. Wang, J. Xu, J. Zhao, Z.-D. Gao and Y.-Y. Song, Chem. Eng. J. **2023**, 476, 146329.
